# The androgen receptor/filamin A complex as a target in prostate cancer microenvironment

**DOI:** 10.1038/s41419-021-03402-7

**Published:** 2021-01-26

**Authors:** Marzia Di Donato, Alice Zamagni, Giovanni Galasso, Erika Di Zazzo, Pia Giovannelli, Maria Vittoria Barone, Michele Zanoni, Roberta Gunelli, Matteo Costantini, Ferdinando Auricchio, Antimo Migliaccio, Anna Tesei, Gabriella Castoria

**Affiliations:** 1 Department of Precision Medicine, University of Campania ‘L. Vanvitelli’- Via L. De Crecchio, 7- 80138 Naples, Italy; 2 Biosciences Laboratory, Istituto Scientifico Romagnolo per lo Studio e la Cura dei Tumori (IRCCS), Via P. Maroncelli, 40- 47014 Meldola, Forlì, Italy; 3 Department of Translational Medical Science, University of Naples ‘Federico II’- Via S. Pansini, 5- 80131 Naples, Italy; 4 Department of Urology, Morgagni Pierantoni Hospital, Forlì, Italy; 5 Pathology Unit, Morgagni Pierantoni Hospital, Forlì, Italy

**Keywords:** Prostate cancer, Cancer microenvironment

## Abstract

Prostate cancer represents the major cause of cancer-related death in men and patients frequently develop drug-resistance and metastatic disease. Most studies focus on hormone-resistance mechanisms related to androgen receptor mutations or to the acquired property of prostate cancer cells to over-activate signaling pathways. Tumor microenvironment plays a critical role in prostate cancer progression. However, the mechanism involving androgen/androgen receptor signaling in cancer associated fibroblasts and consequences for prostate cancer progression still remains elusive. We now report that prostate cancer associated fibroblasts express a transcriptional-incompetent androgen receptor. Upon androgen challenging, the receptor co-localizes with the scaffold protein filamin A in the extra-nuclear compartment of fibroblasts, thus mediating their migration and invasiveness. Cancer-associated fibroblasts move towards epithelial prostate cancer cells in 2D and 3D cultures, thereby inducing an increase of the prostate cancer organoid size. Androgen enhances both these effects through androgen receptor/filamin A complex assembly in cancer-associated fibroblasts. An androgen receptor-derived stapled peptide, which disrupts the androgen receptor/filamin A complex assembly, abolishes the androgen-dependent migration and invasiveness of cancer associated fibroblasts. Notably, the peptide impairs the androgen-induced invasiveness of CAFs in 2D models and reduces the overall tumor area in androgen-treated 3D co-culture. The androgen receptor in association with β1 integrin and membrane type-matrix metalloproteinase 1 activates a protease cascade triggering extracellular matrix remodeling. The peptide also impairs the androgen activation of this cascade. This study offers a potential new marker, the androgen receptor/filamin A complex, and a new therapeutic approach targeting intracellular pathways activated by the androgen/androgen receptor axis in prostate cancer-associated fibroblasts. Such a strategy, alone or in combination with conventional therapies, may allow a more efficient treatment of prostate cancer.

## Introduction

Prostate cancer (PC) represents the most common cancer in men over the age of 50 and skills currently available for its treatment include active surveillance, beam therapy, radical prostatectomy and pharmacologic approaches, mainly the androgen deprivation therapy (ADT; refs. ^1,2^). ADT still represents the standard treatment for PC patients, since androgens influence PC initiation and progression. ADT inhibits, indeed, PC growth and progression. Despite considerable efforts in the development of new androgen/androgen receptor (AR) antagonists, such as abiraterone and enzalutamide, current treatments often fail to achieve long-term efficacy and most patients relapse and develop castrate-resistant PC (CRPC), frequently characterized by metastatic spreading^3^.

Tumor microenvironment is made up of a variety of stromal and inflammatory cells. Among them, cancer-associated fibroblasts (CAFs) are recruited by cancer cell-secreted factors, thereby participating in the cross talk with tumor cells. Once ‘activated’, CAFs reorganize the structure and composition of the connective tissue by depositing extracellular matrix (ECM) and releasing cytokines, as well as growth factors that remodel ECM, increase tumor stiffness and induce growth, invasiveness and drug-resistance of transformed epithelial cells^4^. Prostate CAFs enhance the gland transformation and promote PC progression^5–7^. In addition, they constitute a ‘niche’ that sustains the function of cancer stem cells (CSCs), thus promoting metabolic re-programming of PC cells or their epithelial–mesenchyme transition (EMT; refs. ^8,9^) and metastatic spreading^5,10,11^.

Immunohistochemistry (IHC) analysis has shown that AR expression is very high in non-malignant stroma, as compared to PC stroma^11–13^ and significant levels of the receptor have been detected in CAFs from human PC specimens^14^. Thus it is largely recognized that prostate CAFs express AR^15^, although its role and function in PC is still conflicting^15^. Stromal AR induces prostatic intraepithelial neoplasia (PIN) or metastatic events^16,17^. It might direct fibroblasts towards PC epithelial cells upon a local increase of androgen levels, as frequently occurs in PC^18^. Thereafter, the stromal receptor might induce changes in the tumor microenvironment composition and stimulate the release of growth factors, thus enabling metastatic events^19^.

We previously reported that normal primary or immortalized fibroblasts and fibro-sarcoma cells express low, but significant levels of AR. Upon androgen challenging, these cells undergo migration as a consequence of a bipartite AR/filamin A (FlnA) complex assembly in the extra-nuclear cellular compartment^20–22^. Such complex also regulates the neuritogenesis induced by androgens or nerve growth factor (NGF) in neuronal cells^23^, as well as the motility induced by NGF in PC-derived LNCaP cells^24^.

We now report that most of prostate CAFs from human specimens analyzed express appreciable amounts of AR. The androgen-triggered AR/FlnA complex drives migration and invasiveness of CAFs, as an AR-derived stapled peptide, specifically preventing the AR/FlnA complex assembly in androgen-treated CAFs, inhibits these responses. Similar findings are observed by siRNA FlnA experiments, thus reinforcing the role of AR/FlnA complex in androgen-induced motility and invasiveness in CAFs. CAFs are recruited by PC epithelial cells in in vitro 2D co-culture experiments and mediate a significant increase of PC organoid area, through ECM remodeling. Hormone stimulation enhances both these effects, which are drastically inhibited by the AR/FlnA complex interfering peptide.

In sum, the potential role of signaling activated by the androgen/AR axis that impinges on AR/FlnA complex assembly is here investigated in CAFs. Targeting the AR/FlnA complex by a stapled peptide offers a potential new strategy in PC therapy.

## Materials and methods

### Chemicals, stapled peptide synthesis and constructs

R1881 and dihydro-testosterone (DHT; both from Sigma-Aldrich St. Louis, MO, USA) were used at 10 nM. Enzalutamide (Selleck-chem, Munich, Germany) was used at 10 μM. Unless otherwise stated, enzalutamide was added 30 min before hormonal stimulation. The stapled peptide Rh-2025u was designed and synthesized as reported^21^. It was used at 10 nM and added 30 min before hormonal stimulation. Briefly, Rh-2025u peptide was synthesized by 9-fluorenylmethoxycarbonyl (Fmoc)/ tert-butyl solid-phase method with an Applied Biosystem peptide synthesizer A431. The peptide was assembled on TentaGel R RAm resin and coupling reactions were conducted using the HBTU-HOBt method. N-terminal primary amines were acetylated^21^ and Fmoc-S5-OH was coupled manually in 3-fold molar excess in the presence of HBTU, HOBT, and diisopropylethylamine (DIEA). The ring-closing metathesis reaction was performed on the N-terminal acetylated peptide while still on the solid support^21^. Cleavage of peptide from the resin was achieved with a trifluoroacetic acid/water/triisopropylsilane mixture (92.5/5/2.5, v/v) for 3 h. After the resin had been removed by filtration, the filtrate was concentrated and crude peptides were precipitated with diethyl ether. Crude peptide was purified using reversed phase high-performance liquid chromatography (RP-HPLC) on a preparative C4 column (BioAdvantage Pro 300, Thomson Liquid Chromatography) with a water/acetonitrile solvent system containing trifluoroacetic acid. Purified stapled peptide was characterized by matrix-associated laser desorption ionization time-of-flight mass spectrometry (MALDI micro MX, Waters, Milford, Massachusetts, MA, USA) and RP-HPLC on an analytical C18 column (Eclipse XDB-C18, Agilent, Santa Clara, CA, USA). The purity of the peptide was found to be >95%.

cDNA encoding for the wild-type human AR (hAR) was in pSG5^25^. The 3416 construct was cloned in the *Nhe*I site in pTK-TATA-Luc^26^.

### Cell cultures

All the cell lines here used were from Cell Bank Interlab Cell Line Collection (ICLC- Genova-Italy) and authenticated by the supplier for DNA profile by Short Tandem Repeat (STR) analysis. The cells were maintained at 37 °C in humidified 5% CO_2_ atmosphere. Media and supplements were from Gibco (Thermo Fisher Scientific; Waltham, MA USA). PC-derived fast-growing LNCaP cells were cultured in RPMI-1640 supplemented with 10% fetal bovine serum (FBS), penicillin (100 U/ml), streptomycin (100 U/ml), glutamine (2 mM), sodium pyruvate (1 mM) and non-essential amino acids (0.1 mM). Three days before stimulation, LNCaP cells were made quiescent using phenol red-free RPMI-1640 medium containing 10% charcoal-stripped serum (CSS), penicillin (100 U/ml), streptomycin (100 U/ml) and 0,02 U/l insulin. PC-derived PC3 and DU145 cells were cultured and made quiescent as reported^27^. Breast cancer-derived MCF-7 and T47D cells were grown as described^20,21^. Primary human normal fibroblasts (HNFs; from Invitrogen Carlsbad, California, USA) were cultured according to manufacturer’s instructions. Cell lines were routinely monitored for Mycoplasma contamination and expression of steroid receptors. Hormone responsiveness of LNCaP, MCF-7 and T47D cells was evaluated by BrdU incorporation analysis. Primary prostate CAFs were obtained from PC patient biopsies. Informed consent was obtained for experimentation in human subjects. The ethical Committees approved the study (IRST and Area Vasta Romagna; IRST B.072; 22/03/2017 and PC-StAR Project; University ‘L. Vanvitelli’ B.684; 18/12/2017). PC grade was identified using histopathological analysis of stained frozen sections by Gleason’s score. Fresh tissue fragments adjacent to PC were cut into small fragments. CAFs were separated from epithelial cells after enzymatic digestion^5^ and plated in six-well dishes. Cells were cultured in DMEM supplemented with 10% FBS, penicillin (10 U/ml), streptomycin (100 μg/ml), glutamine (2 mM), insulin (5 μg/ml), transferrin (5 μg/ml) and amphotericin B (2 μg/ml). They were maintained at 37 °C in humidified 5% CO_2_ atmosphere. After reaching confluence, CAFs were expanded and used for no more than 4–5 passages. They were screened with anti-AR, as well as anti-cytokeratin antibodies to confirm the absence of contaminating epithelial cells and anti-vimentin antibody to confirm their mesenchyme nature. Table 1 resumes the data obtained in CAFs from PC patients with different Gleason’s score. About 83% of the analyzed lysates expressed AR. CAFs prepared from the patients indicated in Table 2 were then characterized for the presence of vimentin, α-smooth muscle actin (α-SMA), fibroblast activation protein (FAP), cytokeratin, as well as AR, estrogen receptor alpha (ERα) or progesterone receptor (PR). AR-positive or AR-negative CAFs were pooled and made quiescent using phenol red-free DMEM containing 0.1% CSS, penicillin (100 U/ml), streptomycin (100 U/ml), insulin (5 μg/ml), transferrin (5 μg/ml), and amphotericin B (2 μg/ml). Unless otherwise indicated, CAFs were maintained in this medium for 24 h and used.

**Table 1:** AR expression in CAFs from PC patients.

Gleason’s score	Sample’s number	AR expression	Cytokeratin expression	Vimentin expression
6	13	12/13	0/13	13/13
7	12	10/12	0/12	12/12
8	4	4/4	0/4	4/4
9	6	3/6	0/6	6/6
10	1	1/1	0/1	1/1
	36	30/36	0/36	36/36

**Table 2:** Biochemical properties of CAFs from PC patients.

Patient	Gleason’s score	AR	ERα	PR	α-SMA	FAP
#1	7	+	−	−	+	+
#2	6	+	−	−	+	+
#3	6	+	−	−	+	+
#4	7	+	−	−	+	+
#5	7	+	−	−	+	+
#6	7	+	−	−	+	+
#7	7	+	−	−	+	+
#8	7	−	−	−	+	+
#9	7	+	−	−	+	+
#10	7	+	−	−	+	+
#11	7	+	+	−	+	+
#12	7	−	+	−	+	+
#13	7	+	−	−	+	+
#14	9	−	+	−	+	+
#15	7	+	+	−	+	+
#16	6	+	−	−	+	+
#17	8	+	−	−	+	+

### Transfection, transactivation and siRNA experiments

CAFs were transfected with 1 μg of purified pSG5-hAR-expressing plasmid, using Lipofectamine 2000 (Invitrogen). Control cells were transfected with the empty pSG5 plasmid. After 6 h, transfected cells were made quiescent. In AR nuclear translocation analysis, quiescent cells were left un-stimulated or stimulated for 1 h with 10 nM R1881. In AR transactivation assay, CAFs were co-transfected with purified 3416-pTK-TATA-Luc (4 μg) and left un-stimulated or stimulated for 24 h with the indicated concentrations of R1881. The luciferase activity was analyzed as reported^20^. FlnA siRNA was done^22^, using a 3 target-specific 20-25nt si RNAs pool (Santa Cruz, Dallas, TX, USA). Non-targeting siRNA, containing a scrambled sequence, was from Santa Cruz. CAFs were co-transfected with siRNA Alexa Fluor 488 to help identify the transfected cells. siRNA was transfected in growing CAFs, using Lipofectamine 2000. Optimem/DMEM (50% v/v) medium, containing 10% serum, was used in siRNA transfection. Six hours later, the medium was discarded and transfected CAFs were washed twice using phenol red-free DMEM. The cells were made quiescent for 36 h and used. LNCaP cells were transfected with purified 3416-pTK-TATA-Luc (4 μg), using Lipofectamine 2000. After 6 h, transfected cells were made quiescent, then left un-stimulated or stimulated for 24 h with 10 nM R1881. The luciferase activity was finally analyzed^20^. The plasmid pEGFP-C1 (Addgene; Watertown, MA, USA) was used to generate GFP-stable LNCaP cells. Transient transfection was done using the calcium phosphate-DNA co-precipitating method. Cells at exponential growth-phase were harvested and re-plated at a density of 1 × 10^6^ cells in a 100 mm dish 20 h before the transfection. Cell medium was replaced 2 h before the transfection. A mix of 2.5 M CaCl_2_ (Sigma-Aldrich) and pEGFP-C1 plasmid (15 μg) was added to 2× HEPES buffered saline (140 mM NaCl, 1.5 mM Na_2_HPO_4_, 50 mM HEPES, pH 7.05; Sigma-Aldrich) at 1:1 ratio. The mixture was then added to the plated cells, which were incubated at 37 °C for 6 h and then shocked by addition of 10% glycerol to the medium for 2 min at 37 °C. After a wash with PBS, the glycerol solution (Sigma-Aldrich) was replaced with fresh medium and cells were incubated for additional 2 days. The PC3 cells constitutively expressing GFP were established using the plasmid pEGFP-C1 (5 μg). Cells were transfected using Lipofectamine2000. G418 (Sigma-Aldrich) was used as a selection marker. LNCaP and PC3 cells were splitted into 12-well plates with selecting culture medium containing increasing (from 1000 to 1500 µg/ml) concentration of G418. The selection medium was replaced every 2–3 days. After 7–14 days, cell death was monitored by phase contrast and immunofluorescence (IF) microscopy to identify the GFP-expressing cells. After 8 weeks, the G418-resistant cell clones expressing GFP were selected.

### Cytoskeleton analysis, BrdU incorporation, immunofluorescence (IF), and confocal microscopy

Cytoskeleton changes and BrdU incorporation were analyzed by IF as reported^20,28^. Endogenous AR was visualized using diluted (1:100) rabbit polyclonal antibody against the N-terminal domain of AR (Ab-2; Neomarkers; Portsmouth, New Hampshire, USA). Diluted (1:200 in PBS containing 0.2% bovine serum albumin) anti-rabbit fluorescein isothiocyanate-conjugated antibody (Jackson Laboratories, Bar Harbor, Maine, USA) was used as a secondary reagent. Nuclei were stained with Hoechst 33258 (Sigma-Aldrich) and coverslips were inverted and mounted in Mowiol (Sigma-Aldrich). In AR/FlnA co-localization and AR nuclear translocation analysis, the receptor was revealed using the rabbit Alexa Fluor 488-conjugate anti AR antibody (clone D6F11 from Cell Signaling; Danver, MA, USA). FlnA was stained using diluted (1:50 in PBS) goat polyclonal anti-FlnA antibody (Ab11074; Abcam; Cambridge, UK). Goat antibody was detected using diluted (1:300 in PBS) rabbit anti-goat Texas red-conjugated antibody (Abcam). Cells on coverslips were analyzed using a DMLB (Leica; Wetzlar, Germany) fluorescent microscope, equipped with HCX PL Apo ×63 oil and HCX PL Fluotar ×100 oil objectives. Images were captured using DC480 camera (Leica) and acquired using Leica Suite software. AR/FlnA co-localization was analyzed^29^, using a Zeiss (Oberkochen, Germany) LSM 510 laser scanning confocal microscope.

### Migration, invasion and gelatine metallo-proteinase zymography

Migration assay was done^30^, using quiescent CAFs (3 × 10^4^ per well in 150 µl of cell medium) in collagen-pre-coated Boyden’s chambers with 8 μm polycarbonate membrane (Corning; Corning, NY, USA). The indicated stimuli were added and cytosine arabinoside (Sigma-Aldrich; at 50 µM final concentration) was included in cell medium to inhibit cell proliferation. Cells were allowed to migrate for 7 h. Invasion assay was done^30^, using quiescent CAFs (5 × 10^4^ per well in 200 µl of cell medium) in Boyden’s chambers with 8 μm polycarbonate membrane (Corning) pre-coated with growth factor-reduced and phenol red-free Matrigel (0,6 mg/ml; Corning). The indicated stimuli were added together with cytosine arabinoside (50 µM), and cells were allowed to invade for 24 h. Migrating or invading cells were scored as reported^30^. Zymography assay was done using CAFs at 80% of confluence. Cells were made quiescent, left in serum-free media and then un-stimulated or stimulated for 24 h with 10 nM R1881, in the absence or presence of enzalutamide or Rh-2025u. Conditioned media (CM) were collected and centrifuged, while trypsinized cells were counted. CM were normalized to 1 × 10^6^ CAFs. Samples were concentrated using Amicon Ultra-2 centrifugal filter units (UFC200324; Millipore, Burlington, MA, USA), then mixed with Zymogram sample buffer (#1610764; Bio-Rad, Hercules, California, USA). SDS–PAGE (10% acrylamide) was co-polymerized with gelatine (at 0.1%; from Sigma-Aldrich) and electrophoresis was carried at 30 mA. Gel was washed in a renaturation buffer (#1610765; Bio-Rad) and treated for 36 h at 37 °C with the development buffer (#1610766; Bio-Rad). It was fixed, stained with Comassie Brilliant Blue R-250 and de-stained. MMP-2 proteolytic activity appeared as a clear band migrating at approximately 72 KDa on a blue background.

### 2D co-cultures

LNCaP, PC3, and DU-145 cells were seeded in 24-well plates at 70% of confluence. After 48 h, CAFs (5 × 10^4^ cells/ml) were plated on the top of the 24-well plates, in separate Trans-well inserts with 8 μm polycarbonate membrane (Corning) pre-treated with growth factor-reduced and phenol red-free Matrigel (0,6 mg/ml). The indicated compounds were added to the upper and lower chambers. After 18 h, cell invasiveness was analyzed.

### 3D cultures and co-cultures in ECM

Organoids were generated as reported^31^. When used alone, 2 × 10^4^ GFP-stable expressing PC3 cells were mixed in each well with 200 μl of ECM and 50 μl of organoid plating medium^27^. In co-culture experiments, 2 × 10^4^ GFP-stable expressing LNCaP or PC3 cells and 5 × 10^4^ CAFs were mixed with 200 μl of ECM and 50 μl of organoid plating medium in each well. Whatever the experimental condition, ECM was a 1:4 mix of collagen (4,6 mg/ml; rat tail type I; BD Bioscience, San Jose, CA, USA) and phenol-red free growth factor-reduced Matrigel (10 mg/ml; BD Bioscience). The mixture was pipetted in 24-well plate as reported^32^ and allowed to solidify for 45 min at 37 °C, before the addition of 400 μl organoid plating medium. It was made as reported^33^ using phenol red-free DMEM/F12 medium, containing 7% CSS, penicillin (100 U/ml), streptomycin (100 U/ml), diluted GlutaMAX 100X (Gibco), 10 mM Hepes, B27 supplement (50× stock solution; Thermo Fisher Scientific), 1 M nicotinamide (Sigma-Aldrich), 500 mM N-acetylcysteine (Sigma-Aldrich) and 10 μM Y-27632 (Millipore). After 3 days, the organoid-plating medium was replaced with a similar medium in the absence of N-acetylcysteine and Y-27632^27^. At the 4th day, organoids were untreated or treated with the indicated compounds. The medium was changed every 3 days. Different fields were analyzed using DMIRB Leica (Leica) microscope equipped with C-Plan ×40 or HCX PL Fluotar ×63 objectives (Leica). Phase-contrast and IF microscopy images were acquired using a DFC 450C camera (Leica). Merged images were generated using the Application Suite Software (Leica). The relative organoid size was calculated using the same software and expressed as a fold increase over the basal organoid size, which was measured at 3th day.

### ELISA analysis

LNCaP, PC3, and DU-145 (80 × 10^3^) cells were seeded in 12-well plates and made quiescent. After 48 h, cell culture media were collected and used to assay DHT, testosterone, EGF and β-NGF, according to the manufacturer’s instructions. DHT (KA1886; Abnova; Taipei, Taiwan), testosterone (KGE010; R&D systems; Minneapolis, USA), human EGF (KHG0061; Invitrogen) and human β-NGF (EHNGF; Thermo Fisher Scientific) ELISA kits were used. Data were analyzed using the curve-fitting statistical software Graph Prims Pad.

### DNA isolation, polymerase chain reaction (PCR), sequencing and data analysis

Genomic DNA was extracted from CAFs derived from two different patients (about 1 million cells/patient), using QIAamp DNA Blood Mini kit (Qiagen, Hilden, Germany). DNA purity and concentration were measured by Qubit 2.0 (Thermo Fisher Scientific), using Qubit 1× dsDNA high sensitivity assay kit. hAR coding region (NM_000044) was amplified in 9 amplicons (between 300 and 900 nt in size). Each amplicon includes one exon, except for the exon 1 that was divided in 2 overlapping regions (1A and 1B), because of its big size (1616 nt). Primers were designed to amplify all the exons at the same PCR conditions, also including 20 nt at the splicing junctions. The exon1 needed a primer annealing exactly at its 5’ junction. PCR was done from 50 ng of the genomic DNA, using the AmpliTaqGold (Thermo Fisher Scientific).

**Table:** 

Primer	Fw	Rev	
AR_Ex1A	CGACTACCGCATCATCACAG	GCTCCAACGCCTCCACAC	
AR_Ex1B	GCACTTCGACCATTTCTGAC	GAACACAGAGTGACTCTGC	
AR_Ex2	GAGCAATGAATAATAGTCATTTATGCC	CTCTATTTCTGAGATGATAAAATCC	
AR_Ex3	CTGTTCTAGAAATACCCGAAG	GAAAGGGTCAGCCTGTGTC	
AR_Ex4	GTTGCATTGTGTGTTTTTGACC	GGTCCATAGGAGCGTTCAC	
AR_Ex5	CAGGGACTCAGACTTAGC	CTTCACTGTCACCCCATCAC	
AR_Ex6	GGATGGCAATCAGAGACATTC	GCTTTTCCCTAATAATGTTTTAATGG	
AR_Ex7	GTGGTCAGAAAACTTGGTGC	GTGCCAGACTCTAGAGAAG	
AR_Ex8	GACCAAAAATCAGAGGTTGGG	GAGGAGTAGTGCAGAGTTATAAC	

The 9 amplicons were analyzed by Sanger’s sequencing. PCR products were transferred to a BigDye Terminator v3.1 Cycle Sequencing Kit (Thermo Fisher Scientific) mixture for sequencing reaction, after Illustra ExoProStar 1-STEP Kit purification (GE Healthcare, Chicago, Il, USA), and run into a capillary electrophoresis system (3500xL Dx Genetic Analyzer; Thermo Fisher Scientific). Additional primers were designed to better define 3’ and 5’ junction of exon 1. Electropherograms of each amplicon were analyzed using Mutation Surveyor v5.1.0 (Softgenetics, State College, PA, USA). DNA sequences are deposited in unstructured repository Figshare: (10.6084/m9.figshare.13317575.v1; 10.6084/m9.figshare.13317584.v1).

### Lysates, immune-precipitation (IP), co-immune-precipitation (Co-IP), Rac pull-down assay and Western blot (WB)

All performed as reported^34^, using the following reagents: the mouse monoclonal anti-PR (6A1; Cell Signaling, Beverly, MA, USA), anti-cytokeratin (C11; Santa Cruz), anti-αSMA (CGA7; Santa Cruz), anti-FAP (SS-13; Santa Cruz), anti MT-MMP-1 (SC-373908; Santa Cruz), anti-tubulin (Sigma-Aldrich), anti-FAK and anti P-Tyr397 FAK (both from BD Bioscience) antibodies; the rabbit polyclonal anti-AR (N-20; Santa Cruz), anti-ERα (HC-20; Santa Cruz), anti-vimentin (H-84; Santa Cruz), anti-FlnA (4762S; Cell Signaling) and anti-integrin β1 (Ab1952; Millipore) antibodies. The mouse monoclonal anti-AR (441; Santa Cruz) antibody was used to immune-precipitate and detect AR in Co-IP experiments. Rac pull-down assay was done^20^, using the Rac activation kit (Upstate Biotechnology, Burlington, MA, USA). The ECL system (GE Healthcare) was used to reveal immune-reactive proteins.

### Statistical analysis and data availability statement

Experiments were performed in triplicate and data presented as the mean ± standard deviation. The differences between values observed after the various treatments were analyzed using the paired Student’s *t* test. *p* value < 0.05 was considered significant. The data generated during this study are available from the corresponding author on reasonable request.

## Results

### Expression of AR in prostate CAFs

**Fig. 1:**
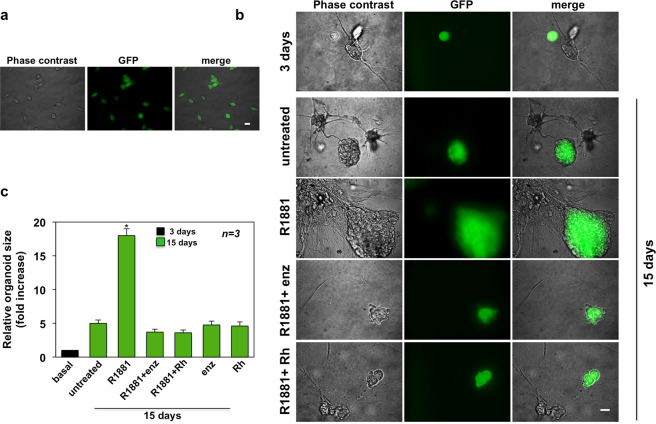
LNCaP-CAFs organoid growth: effect of Rh-2025u peptide. LNCaP cells stably expressing GFP were used. In **a**, cells were analyzed by phase contrast (left section) and fluorescence microscopy (middle section). Merged images are shown in the right section. Scale bar, 10 μm. AR-positive CAFs from different patients (Table 2) were pooled and used. In **b** and **c**, GFP-LNCaP cells in ECM were co-cultured with CAFs for 3 days (basal condition). Cells were then left unchallenged or challenged with 10 nM R1881, in the absence or presence of 10 nM Rh-2025u peptide (Rh), or 10 μM enzalutamide (enz). Control cells were treated with the peptide or enzalutamide, alone. In **b**, the organoid size was monitored for 15 days and representative images by phase-contrast (left section), or fluorescence microscopy (middle section) were captured and shown. The corresponding merged images are shown in the right section. Scale bar, 100 μm. In **c**, the organoid size was calculated using the Leica Suite software under basal conditions (3 days) or in cells left unstimulated or stimulated for 15 days, in the absence or presence of the indicated compounds. It was finally expressed as a fold increase in the relative LNCaP-CAFs organoid size. *n* represents the number of experiments. Means and SEM are shown. *p < 0,01, as compared to basal or untreated cells.

CAFs from patients # 1–17 (Table 2) were prepared. Their purity was confirmed by the absence of the epithelial marker, cytokeratin, and presence of the mesenchyme marker, vimentin (Fig. 1S, a). CAFs, but not human normal fibroblasts (HNFs), expressed α-SMA and FAP (Fig. 1S, b), which are indicative of the fibroblast activated-phenotype^35^. Expression of AR was analyzed by WB technique. Most of the analyzed CAFs (Table 2) expressed low, but detectable levels of AR, as compared to those expressed in LNCaP cells (Fig. 1S, c). Various mesenchyme and mesenchyme-transformed cells express low levels of AR^20–22^. Of note, low levels of stromal AR correlate with PC bad prognosis^36^. The anti-AR antibody used (raised against the NH2-terminal sequence) reacted with a band migrating at 110 KDa, and in some lysate samples, including those prepared from LNCaP cells, it also recognized a minor band migrating at 95 KDa (Fig. 1S, left panel in c). This latter immune-reactive band might represent an AR-truncated isoform or a proteolytic product of the receptor^37^. AR might undergo degradation through the MDM2 (mouse double minute 2 homolog)-mediated ubiquitin–proteasome pathway^38^. In addition, other E3 ligases, such as NEDD4 (neural precursor cell expressed developmentally down-regulated protein 4), CHIP (C-terminus of Hsp70-interacting protein), and SKP2 (S-Phase Kinase-Associated Protein 2) might also regulate AR proteolysis in LNCaP cells^39,40^. Although ERα has been revealed by immune-histochemical (IHC) analysis in 60% of CAFs from PC specimens^41^, we detected the receptor in only 20% of the analyzed CAFs (Fig. 1S, c). Differences related to the models (frozen tissues or cultured cells), or anti-ER antibodies used might allow these discrepancies^42^. In addition, we did not detect PR in lysate proteins from CAFs. ERα and PR were, instead, detected in MCF-7or T47D cell lysates and used as positive controls for ERα or PR expression, respectively (Fig. 1S, c).

### AR-positive CAFs from PC patients increase the size of LNCaP and PC3 organoids: inhibitory effect of the Rh-2025u stapled peptide

**Fig. 2:**
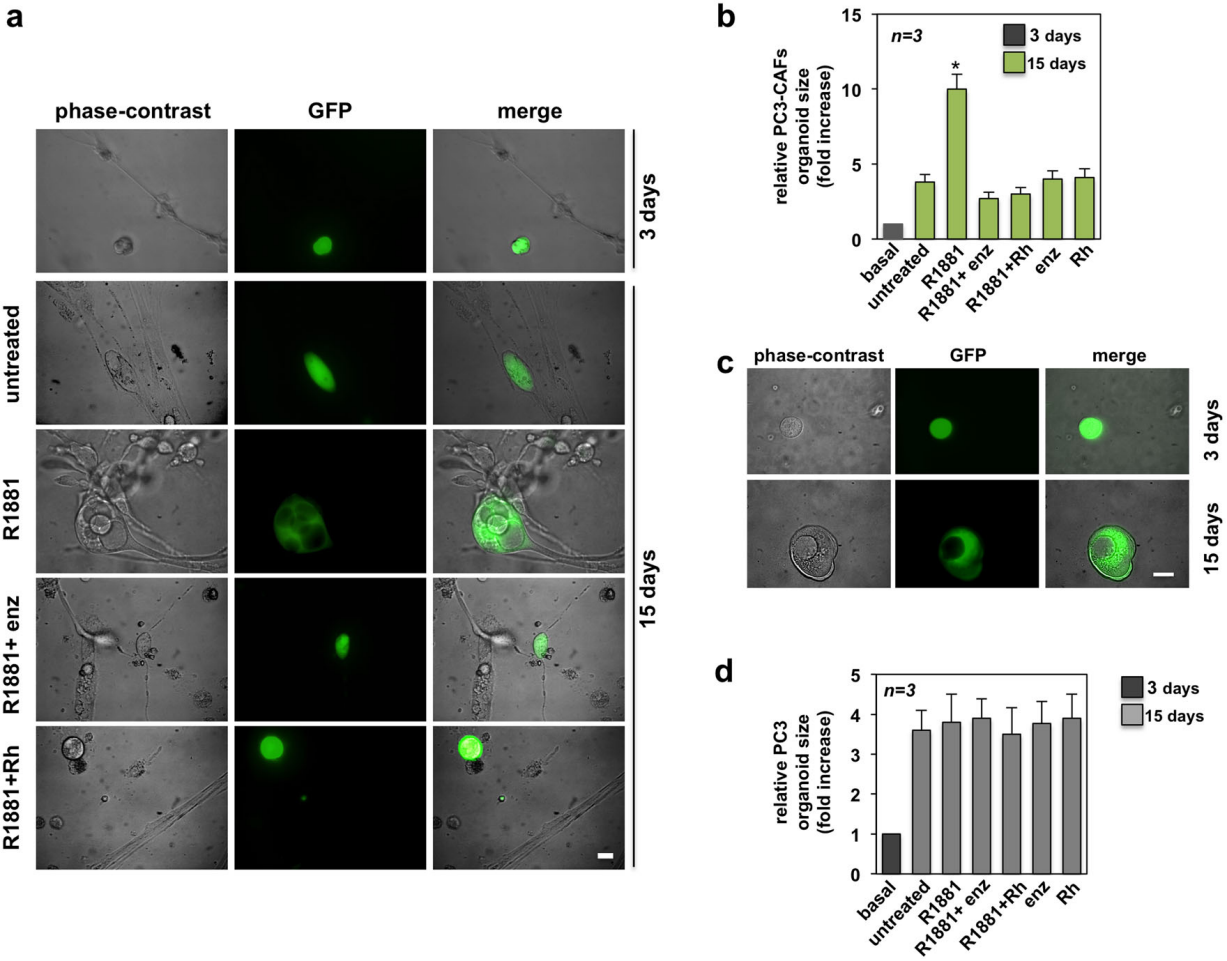
PC3-CAFs organoid growth: effect of Rh-2025u peptide. AR-negative PC-derived PC3 cells stably expressing GFP were cultured in ECM, in the presence of pooled AR-positive CAFs from different patients (Table 2), for 3 days (basal condition). Cells were then left unchallenged or challenged for 15 days with 10 nM R1881, in the absence or presence of 10 nM Rh-2025u peptide (Rh), or 10 μM enzalutamide (enz). Control cells were treated with the peptide or enzalutamide, alone. In **a**, the organoid size was monitored by phase-contrast (left section), or fluorescence microscopy (middle section). Merged images are shown in the right section. Scale bar, 100 μm. In **b**, the organoid size was calculated as described in the legend to Fig. 1 and expressed as fold increase in the relative PC3-CAFs organoid size. In **c** and **d**, GFP-PC3 cells were cultured in ECM, in the absence of CAFs. In **c**, the organoid size was monitored on 3 and 15 days and representative images by phase-contrast (left section) or fluorescence microscopy (middle section) were captured and shown. The corresponding merged images are shown in the right section. Scale bar, 100 μm. In **d**, GFP-PC3 cells in ECM were cultured for 3 days (basal condition). The cells were then left unchallenged or challenged with 10 nM R1881, in the absence or presence of 10 nM Rh-2025u peptide (Rh), or 10 μM enzalutamide (enz). Control cells were treated with the peptide or enzalutamide, alone. The organoid size was calculated as described in the legend to Fig. 1 and expressed as fold induction in the relative PC3 organoid size. In **b** and **d**, *n* represents the number of experiments. Means and SEM are shown. *p < 0,05, as compared to basal or untreated cells.

We next generated miniaturized PC-CAF co-cultures in ECM. To this end, AR-positive CAFs from patients in Table 2 were pooled together to minimize data spread due to inter-individual variation, then co-cultured with LNCaP cells stably expressing GFP (GFP-LNCaP cells; Fig. 1a) in ECM. A 3D structure was observed in GFP-LNCaP cells after 3 days of co-culture with CAFs (Fig. 1b). At that time, the organoids were left untreated or treated with 10 nM of the non-aromatizable androgen R1881^43^, in the absence or presence of 10 μM of the AR-antagonist, enzalutamide^44^, or 10 nM of the stapled peptide, Rh-2025u. This latter compound derives from the AR 622–670 amino acid sequence responsible for interaction of the receptor with FlnA^22,45^. Because of the strong cell permeability, higher affinity for the target and decreased degradation exhibited by the stapled version of peptides, as compared to their un-stapled counterparts^46^, we designed a stapled version of the peptide (ref. ^21^ and Fig. 2S, a). The procedure of peptide synthesis has been previously published^21^ and summarized in Method’s section. At 10 nM concentration, the stapled peptide specifically disrupts the androgen-induced AR/FlnA complex assembly and motility in mouse and HNFs, as well as human fibrosarcoma-derived HT1080 cells^21,22^. It also reverses the androgen-induced AR/FlnA complex assembly, thereby inhibiting the neuritogenesis in PC12 cells^23^. Supplemental Data in Fig. 2S resume the most important properties of this compound. It does not perturb motility and invasiveness induced by serum in AR-positive CAFs (Fig. 2S, b) or PC-derived cells (Fig. 2S, c). Notably, the compound does not function in AR-negative CAFs (Fig. 2S, d) and unlike other inhibitors currently used in PC clinical trials, such as enzalutamide, the peptide exhibits its efficacy at very low (nano-molar) concentration. It only affects the small pool of AR that binds FlnA, leaving unaltered other receptor functions, such as gene transcription in LNCaP cells (Fig. 2S, e).

**Fig. 3:**
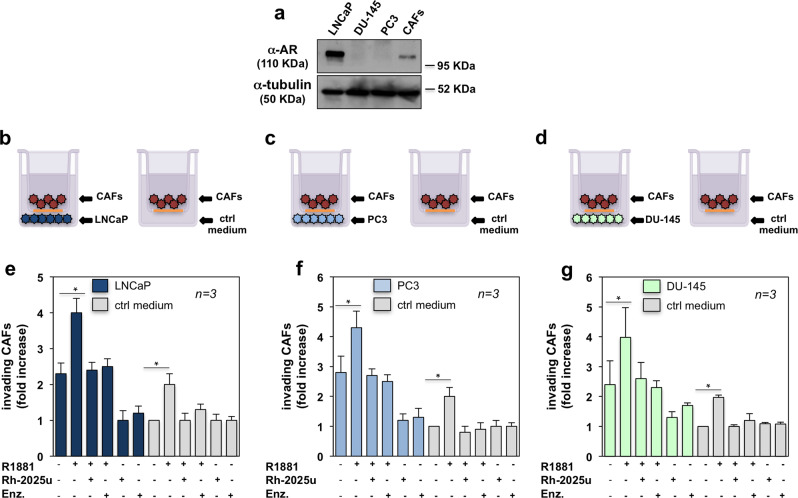
2D co-culture experiments: effect of Rh-2025u peptide. LNCaP cells, PC3 and DU-145 cells, as well as AR-positive CAFs pooled from different patients (Table 2) were used. The upper section in **a** shows the WB of lysates from the indicated cells with anti-AR antibody. The lower section in **a** shows the WB of the corresponding lysate proteins with anti-tubulin antibody, as loading control. Schematic representations in **b**, **c**, and **d** show that AR-positive CAFs were co-cultured with LNCaP cells (left in **b**) or unconditioned medium (ctrl medium; right in **b**), PC3 cells (left in **c**) or unconditioned medium (ctrl medium, right in **c**), DU-145 cells (left in **d**) or unconditioned medium (ctrl medium, right in **d**), using a Trans-well system. In **e**–**g**, cells were left untreated or treated with the indicated compounds. R1881 and Rh-2025u peptide were used at 10 nM. Enzalutamide was used at 10 µM. CAFs were allowed to invade for 16 h, stained and counted as described in “Methods” section. Data are expressed as fold increase in number of invading CAFs. *n* represents the number of experiments. Means and SEM are shown. *p < 0,05.

Changes in dimension and structure of organoids were then monitored for additional 15 days. Phase-contrast microscopy and IF images (Fig. 1b) together with quantification of data (Fig. 1c) show that stimulation with 10 nM R1881 resulted in a significant increase of the LNCaP organoid size and similar findings were observed using 10 nM of the androgen DHT (Fig. 3S, a and b). A slight, but significant stimulation was observed using 2 nM R1881. Of note, R1881 and DHT exhibit equivalent AR binding affinities^47^, but the synthetic androgen R1881 is non-aromatizable. Therefore, we choose to use R1881 throughout the manuscript. The observation that the maximal increase in LNCaP organoid size was detected at 10 nM androgen concentration supports a dose-dependent effect of ligand. Although AR occupancy is almost completely saturated by 2 nM ligand concentrations, the number of receptors, the affinity and on/off rates of AR ligand interactions, as well as the receptor synthesis and degradation might influence the AR activity in target cells^47^ and refs therein. Moreover, we have shown that the optimal AR/FlnA complexation occurs at 10 nM androgens^21–23^. These findings likely account for the ligand concentration-dependent effects here reported.

Enzalutamide and Rh-2025u both abolished the R1881-induced effect on organoid size (Fig. 1b, c), while leaving unaffected this response when used in the absence of hormone (Fig. 1c). Androgen treatment resulted in recruitment of AR-positive CAFs by LNCaP organoid (Fig. 1b). Stromal components, which are not fluorescent, made up a cell network surrounding the LNCaP organoid (marked in green). Here again, enzalutamide or Rh-2025u inhibited these effects.

We next used the AR-negative PC3 cells stably expressing GFP (GFP-PC3). Notably, the PC3 organoid size significantly increased over the untreated level when GFP-PC3 were co-cultured with AR-positive CAFs in the presence of androgen (Fig. 2a, b). Here again, AR-positive CAFs were detected in close proximity of the organoid, when androgen was added. Enzalutamide or Rh-2025u inhibited both the recruitment of CAFs by PC3 organoids, as well as the increase in organoid size (Fig. 2a, b). In the absence of CAFs, we only detected an increase over the basal level (3 days culture) of about 4-folds in organoid size upon 15 days of culture (Fig. 2c, d). Expectedly, hormone addition did not modify the PC3 organoid size, since PC3 cells do not express AR and are insensitive to androgens^27^. Regardless of experimental conditions, neither enzalutamide, nor Rh-2025u peptide modified the organoid size (Fig. 2d).

**Fig. 4:**
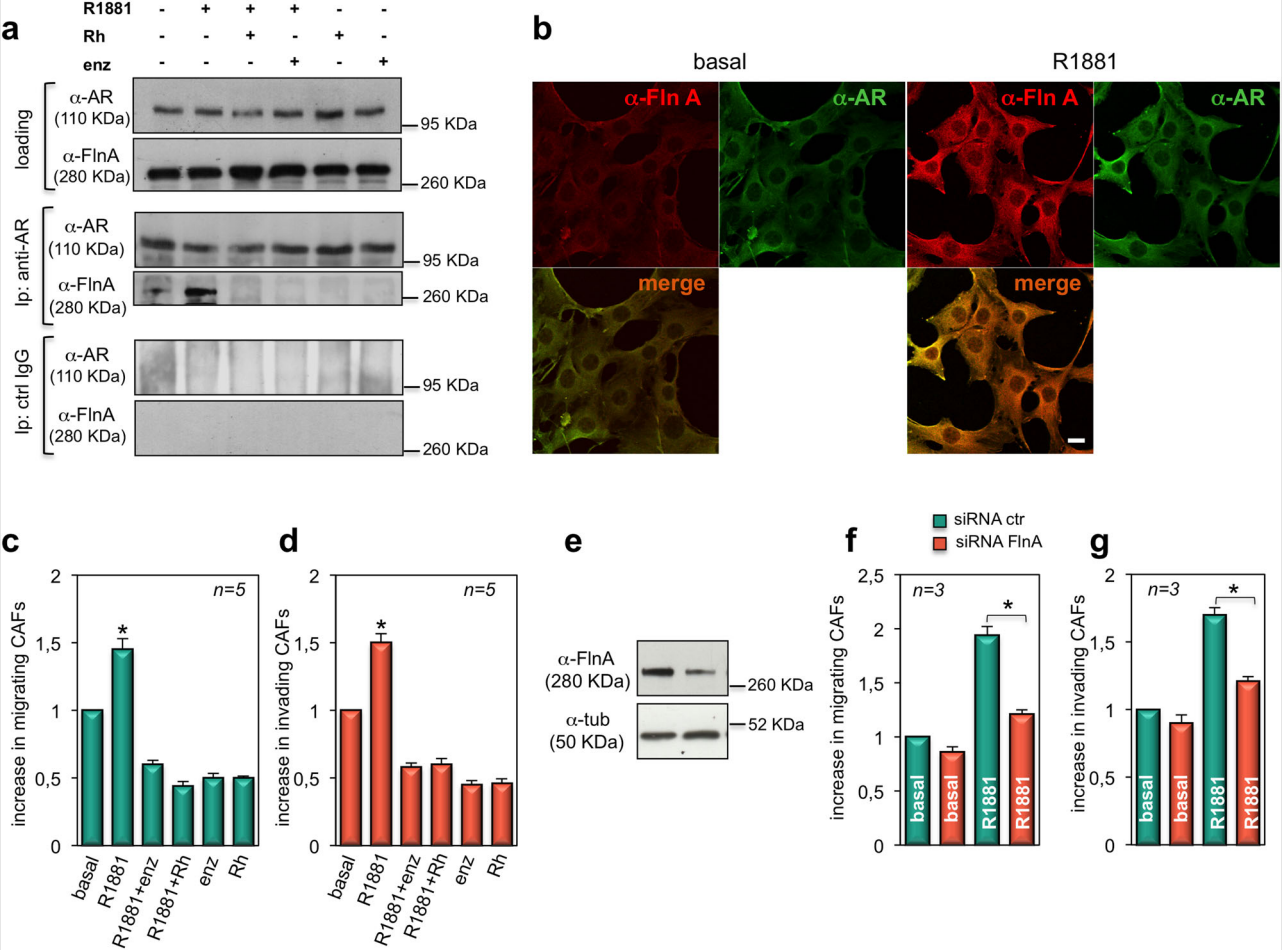
The androgen-triggered AR/FlnA complex assembly and co-localization mediates motility and invasiveness of CAFs. In **a**, AR-positive CAFs pooled from different patients (Table 2) were made quiescent and then left unchallenged or challenged for 10 min with 10 nM R1881, in the absence or presence of the indicated compounds. Enzalutamide (Enz) was used at 10 μM. Ten nM Rh2025u peptide (Rh) was added 30 min before hormonal stimulation. The upper section in **a** shows the WB with the indicated antibodies to reveal lysate proteins (loading), which were then immune-precipitated using the anti-AR antibody (middle section, anti-AR Ab) or control IgG (lower section, ctrl IgG). The WB with the indicated antibodies was done to detect proteins in immune-complex. In **b**, quiescent CAFs from patient #4 (Table 2) were left untreated (basal) or treated for 10 min with 10 nM R1881 (R1881). Cells on coverslips were stained for AR and Fln A. Images captured by confocal microscope show the staining of AR (green) and Fln A (red). Merged images are presented in the right panels. They are representative of three independent experiments. Bar, 5 μm. In **c** and **d**, quiescent CAFs from patient #4 (Table 2) were left unchallenged or challenged with 10 nM R1881, in the absence or presence of the indicated compounds. Enzalutamide (Enz) was used at 10 μM, and Rh2025u peptide (Rh) at 10 nM. Cells were allowed to migrate (**c**) or invade (**d**) for 7 h or 24 h, respectively. They were scored as described in “Methods” section and data were expressed as fold increase. Means and SEMs are shown. ***n*** represents the number of experiments. *p < 0,05 for the indicated experimental points versus the corresponding untreated control (basal). In **e**–**g**, AR-positive CAFs pooled from different patients (Table 2) were used. Growing cells were transfected with siRNA Alexa Fluor 488 along with control siRNA (siRNA ctr) or siRNA FlnA (siRNA FlnA), as described in “Methods” section. After transfection, lysate proteins were analyzed by WB using the indicated antibodies (**e**). CAFs were made quiescent and left in the absence or presence of 10 nM R1881. CAFs were then allowed to migrate (**f**) or invade (**g**) for 7 h or 24 h, respectively. Migrating (**f**) or invading (**g**) CAFs were scored as described in “Methods” section and data expressed as fold increase in number of migrating or invading cells. Results from several independent experiments were collected and analyzed. Means and SEM are shown. *n* represents the number of experiments. *p < 0,05 for the indicated experimental points versus the corresponding untreated control (basal).

Thus AR-positive CAFs robustly increase the PC organoid size upon androgen stimulation, regardless of PC cells used (LNCaP or PC3 cells). The stapled peptide, which perturbs the androgen-induced AR/FlnA complex assembly in CAFs (see the Fig. 4 in this paper), reduces the size of LNCaP and PC3 organoids and impairs the androgen-induced recruitment of AR-positive CAFs by PC organoids. These results make the AR/FlnA complex a putative target in prostate CAFs.

### Recruitment of AR-expressing CAFs from PC patients by LNCaP, PC3 or DU-145 cells in 2D co-culture: inhibitory effect of the Rh-2025u peptide

We next co-cultured the AR-positive LNCaP cells with AR-positive CAFs (pooled from patients indicated in Table 2). Expression of AR was confirmed by WB analysis of the corresponding cell lysates (Fig. 3a). Unconditioned medium for LNCaP cell culture (right inset in Fig. 3b) was used as a control. After co-culture of CAFs with LNCaP cells (left inset in Fig. 3b), cells were left untreated or treated with 10 nM R1881, in the absence or presence of the indicated compounds (Fig. 3e). Co-culture with LNCaP cells stimulated by 2-folds the number of invading CAFs, as compared to the control, unconditioned medium. Androgens enhanced by about 2-folds the recruitment of AR-positive CAFs by LNCaP cells. Rh-2025u and enzalutamide reverted this effect (Fig. 3e). In addition, co-culture with LNCaP cells slightly increased (from 1 to 1,35) the number of invading AR-negative CAFs, as compared to control, unconditioned medium (Fig. 3S, c). Expectedly, AR-negative CAFs were insensitive to R1881 (Fig. 3S, c). The finding that LNCaP cells recruit CAFs under basal conditions might be due to a local increase in androgen^48^, cytokine and growth factor^49^, or nerve growth factor (NGF;^50,51^) levels. Analysis of conditioned medium by ELISA assay indicates that LNCaP cells release appreciable amounts of testosterone and NGF. In contrast, only low amounts of DHT and EGF were detected (Fig. 3S, d). Thus, LNCaP cells might recruit AR-positive CAFs through testosterone release. In turn, stromal cells might convert testosterone into DHT, since they express 5α-reductase (5α-R) enzymes^52^. The weak increase in AR-negative CAF recruitment by LNCaP cells might be caused by NGF release (Fig. 3S, c and d).

We then used AR-negative PC3 cells (Fig. 3a) in co-culture with AR-positive CAFs. Here again, unconditioned medium employed for PC3 cell culture (right inset in Fig. 3c) was used as a control. After co-culture of CAFs with PC3 cells (left inset in Fig. 3c), cells were left untreated or treated with R1881, in the absence or presence of the indicated compounds (Fig. 3f). Co-culture with PC3 cells increased by almost 3-folds the number of invading CAFs, as compared to the control, unconditioned medium. As before stated, such an increase might be related to the paracrine loop between stromal and PC cells. PC3 cells release androgens and NGF, in addition to a very low amount of EGF (Fig. 3S, d). R1881 increased by 1,7 folds the number of invading CAFs and this effect was inhibited by 10 nM Rh-2025u or 10 μM enzalutamide (Fig. 3f). Similar results were observed when AR-positive CAFs were co-cultured with AR-negative DU-145 cells (Fig. 3a, d and g). Thus, by perturbing the AR/FlnA complex assembled in CAFs, the stapled peptide inhibits the androgen-dependent recruitment of CAFs by PC3 or DU-145 cells. The possibility that Rh-2025u acts in PC3 or DU-145 cells is excluded by the finding that the stapled peptide does not function in AR-negative cells (ref. ^21^ and Fig. 2S).

Thus, PC epithelial cells recruit CAFs under basal conditions because of a paracrine regulatory loop. Androgens significantly enhance such effect, which likely requires the assembly of AR/FlnA complex in CAFs.

### The androgen-triggered AR/FlnA complex assembly regulates migration and invasiveness of CAFs: inhibitory effect of the Rh-2025u peptide

Given the data so far presented, we analyzed the AR/FlnA complex assembly in CAFs. In a first attempt, we evaluated by WB the expression of FlnA in CAF lysates derived from different patients (Fig. 4S, a). Thereafter, we surveyed by Co-IP approach the androgen effect on AR/FlnA complex assembly. Lysates from AR-expressing CAFs (pooled from patients in Table 2) were used. Similar amounts of AR or FlnA were detected in CAF lysates, regardless of experimental conditions (upper section in Fig. 4a). Ten minutes challenging with 10 nM R1881 significantly stimulated the association of AR with FlnA in CAFs. Addition of 10 nM Rh-2025u peptide abolished this association and a similar effect was observed using 10 μM enzalutamide. Both compounds did not affect the AR/FlnA complex assembly when used in the absence of hormone (middle section in Fig. 4a). The absence of AR and FlnA in CAF lysates immune-precipitated with control antibodies confirmed the specificity of Co-IP approach (lower section in Fig. 4a).

We then investigated by confocal microscopy the androgen effect on AR/FlnA co-localization in CAFs from patients indicated in Fig. 4S, b. Quantitative analysis from different experiments shows that androgen increased AR/FlnA co-localization ratio by almost 2-fold. Representative images captured from one experiment in CAFs from patient # 4 are presented in Fig. 4b. A significant increase in co-localization ratio between AR (green) and FlnA (red) was observed in the extra-nuclear compartment of androgen-treated CAFs. Control images captured from CAFs stained with secondary antibody alone, in combination with the FITC-conjugated anti AR antibody, show the specificity of FlnA staining (Fig. 4S, c).

We next analyzed the migration (Fig. 4S, d) and invasiveness (Fig. 4S, e) induced by different concentrations of R1881 or DHT in CAFs. Consistent with data in Fig. 3S, both ligands did not significantly affect migration or invasiveness when used at 0,1 nM, while a slight increase was observed at 1 nM ligand concentration. A 2,5-fold increase in both the effects was observed in CAFs challenged with 10 nM androgens, further supporting a dose-dependent response to ligand stimulation. We then evaluated the effect of Rh-2025u peptide on motility and invasion induced by 10 nM R1881 in CAFs from patient # 4. Androgen challenging significantly increased CAF motility (Fig. 4c) and invasiveness (Fig. 4d). Ten nM Rh-2025 peptide or 10 μM enzalutamide inhibited both the effects. Similar results were observed in CAFs from different patients (Table I in Supplemental Data). In summary, androgens enhance AR/FlnA complex assembly and co-localization in extra-nuclear compartment of CAFs. Interference in this complex by the peptide reverses the androgen-induced CAF migration and invasiveness.

At last, we assessed the role of FlnA by siRNA approach. Since primary cells are refractory to siRNA stable transfection^53^, we transiently transfected AR-positive CAFs (pooled from patients in Table 2) with siRNA FlnA and analyzed the effect of this knockdown in androgen-induced migration and invasiveness. The WB analysis shows that FlnA level decreased by about 70 % in siRNA FlnA transfected cells (Fig. 4e). Such knockdown significantly inhibits the androgen-triggered migration (Fig. 4f) and invasion (Fig. 4g) of CAFs. In summary, findings collected in Fig. 4 show that pharmacologic inhibition of AR/FlnA complex by a tailored peptide or somatic FlnA knockdown impair the migratory and invasive phenotype elicited by androgens in AR-expressing CAFs.

### Analysis of AR expressed in CAFs

**Fig. 5:**
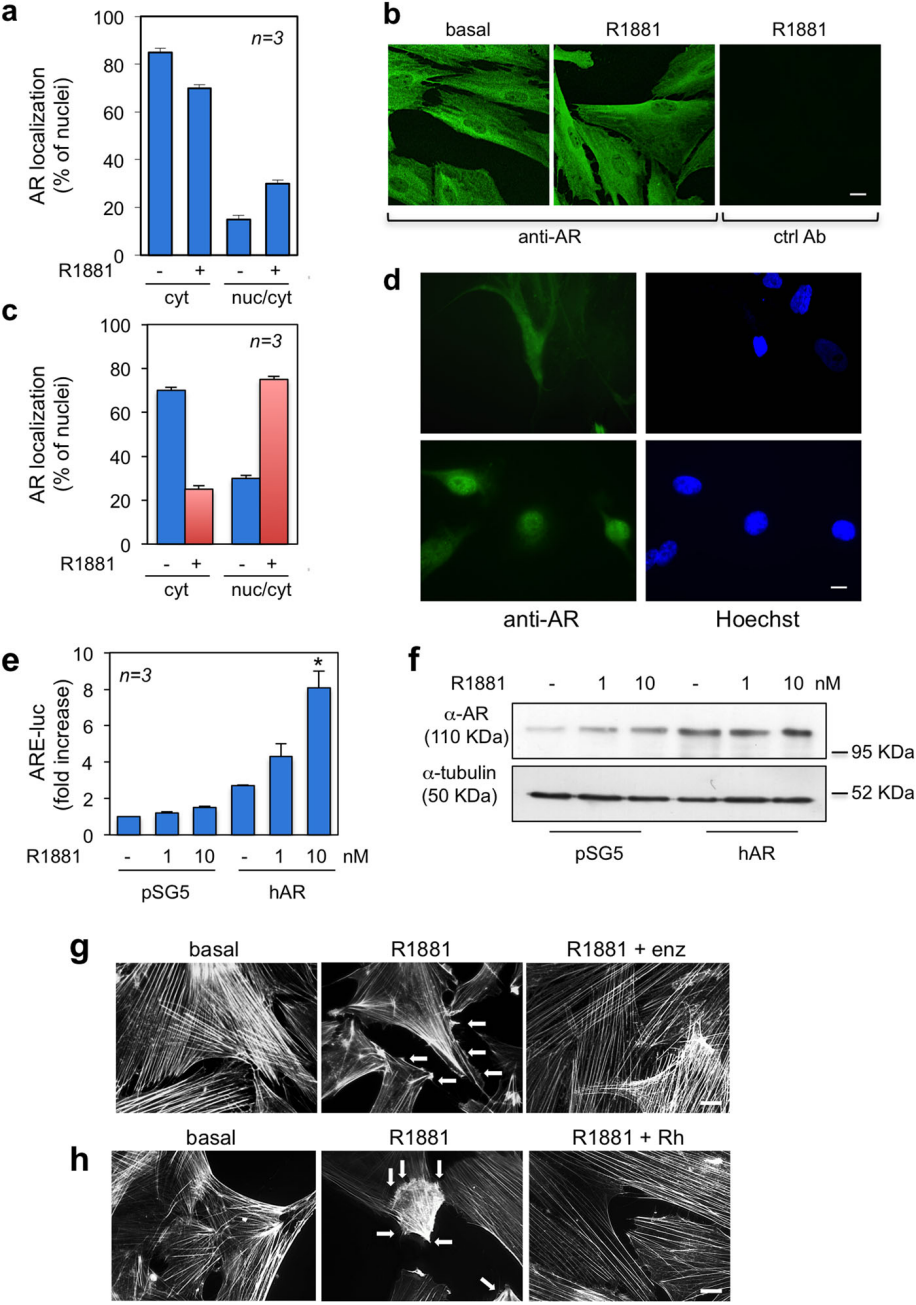
Biological responses mediated by AR expressed in prostate CAFs. AR-positive CAFs from patient #4 (Table 2) were used. In **a**, quiescent CAFs on coverslips were left untreated or treated for 60 min with 10 nM R1881. Cells were stained for AR or nuclei and analyzed by IF, as described in “Methods” section. AR localization was expressed as percentage of cells showing a predominant cytoplasmic (cyt) or nuclear/cytoplasmic (nuc/cyt) AR localization. Representative images captured from one experiment in **a** are shown in panel **b**. In **c**, **d**, CAFs were transfected with hAR expressing plasmid or empty pSG5 plasmid, as control. In **d**, quiescent transfected cells on coverslips were left untreated or treated for 60 min with 10 nM R1881. CAFs were stained for AR or nuclei and analyzed by IF, as described in “Methods” section. The score of CAFs showing a predominant cytoplasmic (cyt) or nuclear/cytoplasmic (nuc/cyt) AR localization was also done and reported in **c**. Representative images were captured and shown in **d**. In **e**, **f** CAFs were transfected, with 3416-luc constructs, in the presence of the control pSG5 or hAR expressing plasmid. Transfected cells were made quiescent and then left unstimulated or stimulated for 24 h with 1 or 10 nM R1881. Luciferase activity was assayed, normalized using β-gal as an internal control and expressed as fold induction (panel **e**). Over-expression of hAR was also evaluated by WB of lysate proteins from CAFs transfected with the empty pSG5 or hAR encoding plasmid (upper section in **f**). The lower section in **f** shows the WB of corresponding CAF lysate proteins with anti-tubulin antibody. In **g** and **h**, quiescent CAFs on coverslips were left unstimulated or stimulated for 20 min with 10 nM R1881, in the absence or presence of 10 nM Rh2025u peptide (Rh; in panel **g**) or 10 μM enzalutamide (enz; in panel **h**). Cells were analyzed for F-actin, as described in “Methods” section. Arrows indicate CAFs protrusions and ruffles. In **b**, **e**, **g**, and **h**, bar, 5 µm. In **a**, **c**, and **e** means and SEM are shown; *n* represents the number of experiments. In **c**, *p < 0,05 for the indicated experimental points versus the corresponding untreated control.

Given the results so far presented, we looked at the biological properties of AR expressed in CAFs. We firstly analyzed by IF the intracellular distribution of AR in CAFs from patient #4 (Table 2). The score of cells exhibiting cytoplasmic AR fluorescence shows that the receptor was mostly unable to translocate into nuclei and was prevalently localized in cytoplasm, regardless of androgen stimulation. A low (<20%) fraction of cells showing nuclear/cytoplasmic AR staining was detected under basal conditions, and a slight increase in nuclear staining, simultaneously with a small decrease in cytoplasm, was observed by 1h androgen challenging of cells (Fig. 5a). Figure 5b show images captured from one experiment presented in panel a. The absence of fluorescence in CAFs stained with the secondary antibody alone also indicates the specificity of our approach (right image in b). Similar data were observed in CAFs derived from different patients (Table II in Supplemental Data). In contrast, over-expression of hAR, which was verified by WB of CAF lysate from patient # 4 (panel f), resulted in a robust nuclear translocation of the receptor by 1h hormone stimulation (c and d). Given these results, we verified the AR transactivation by a reporter assay. CAFs from patient #4 were transfected with androgen-responsive element (ARE)-luc and then left untreated or treated with 1 or 10 nM R1881. Irrespective of hormone concentration, luciferase activity was only slightly increased when CAFs transfected with a control plasmid were stimulated with androgens. Similar data were collected in CAFs from different patients (Table III in Supplemental Data). A weak, but significant increase in luciferase activity was observed upon 1 nM hormone challenging, while a robust ARE-luc activation was detected at 10 nM R1881 stimulation of the CAFs transfected with hAR-encoding plasmid (Fig. 5e, f). Noticeably, CAFs used in our reporter assay express low amounts of AR, as compared to those observed after overexpression of hAR (Fig. 5f). Thus, the failure of AR in mediating a significant androgen-induced gene transcription in CAFs is likely due to low levels of receptor, which do not allow its dimerization and nuclear translocation. In support of this hypothesis, transfection of CAFs with hAR cDNA restored the nuclear translocation (c and d), as well as the genomic functions (e) of AR in androgen-stimulated CAFs.

Changes in the actin cytoskeleton are hallmarks of migrating cells^54^. F-actin images of AR-expressing CAFs from patient # 4 show that 10 nM R1881 induced within 20 min the appearance of ruffles and protrusions (marked by the arrows in Fig. 5g, h). Enzalutamide (g) and Rh-2025u peptide (h) inhibited the R1881-induced cytoskeleton changes. These findings indicate that AR challenging by 10 nM androgens promotes cytoskeleton changes that allow migration and invasiveness of CAFs through the AR/FlnA complex assembly.

### Androgen triggers AR/β1 integrin/MT-MMP-1 complex assembly and MMP-2 release: a mechanism of CAFs invasion through ECM

**Fig. 6:**
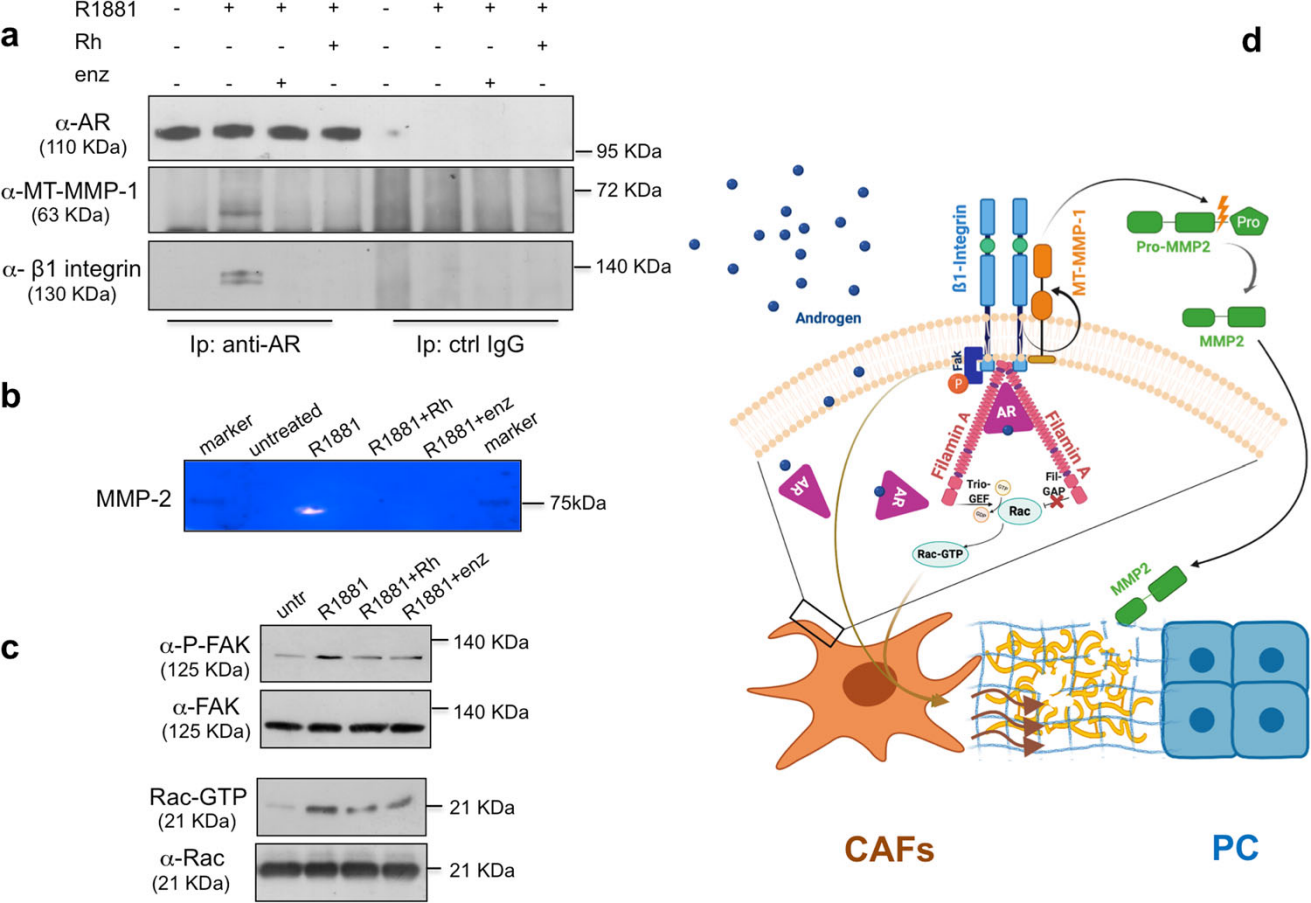
Androgen triggers AR/β1 integrin/MT-MMP-1 complex assembly and MMP-2 release: a mechanism of CAFs invasion through ECM. AR-positive CAFs pooled from different patients (Table 2) were made quiescent. In **a**, cells were left unchallenged or challenged for 10 min with 10 nM R1881, in the absence or presence of 10 μM enzalutamide (enz) or 10 nM Rh2025u peptide (Rh). Lysate proteins were immune-precipitated using the anti-AR antibody (anti-AR) or control IgG (ctrl IgG). The WB with the indicated antibodies was done to detect proteins in immune-complex. In **b**, cells were untreated or treated for 24 h as in **a**. Release of MMP-2 in conditioned media was analyzed by zymography, as described in “Methods” section. In **c**, lysate proteins were analyzed for FAK activation, using the anti-P-Tyr397FAK antibody. The filter was stripped and re-probed using anti-FAK antibody (upper section). In the lower section, proteins from CAFs lysates were used for Rac pull down assay. The WB with anti-Rac antibody revealed the eluted Rac (Rac-GTP). The total amount of Rac expressed in the corresponding CAFs lysates was also detected. The WB for tubulin expression in CAFs lysate proteins was finally done, as loading control. Results in **c** are representative of 3 different experiments. In **d**, androgen stimulation of AR-expressing CAFs triggers the assembly of AR/FlnA/β_1_ integrin complex. Such complex is required to activate FAK and Rac, thereby triggering CAFs motility, on one hand. On the other, the AR/FlnA/β_1_ integrin complex recruits MT-MMP-1, thus activating pro-MMP2 and the consequent release of MMP-2. Protease-mediated ECM remodeling then follows, thus promoting CAFs invasiveness and their recruitment towards PC cells.

At last, we hypothesized that stromal AR mediates changes in ECM composition on hormone challenging. CAFs release the matrix metalloproteinase (MMP)-2 as inactive pro-enzyme (pro-MMP-2) in tumor microenvironment. MT-MMP-1 (also known as MMP-14) activates pro-MMP-2, promotes the release of MMP-2 and allows ECM degradation, as well as cell invasiveness^55^. Such a mechanism involves β1 integrin in PC^56^ and mammary cells^57^. Of note, we previously showed that androgens trigger the recruitment of β1 integrin to AR/FlnA complex in various cell types, including fibroblasts^21–23^. As the stapled peptide significantly perturbs the androgen-triggered AR/FlnA/β1 integrin complex assembly^21–23^, we thought that complex disassembly by peptide would inhibit MMP-2 release by CAFs and the consequent ECM remodeling. Findings in Fig. 6 address this issue, since they show that β1 integrin co-immunoprecipitated with MT-MMP-1 and AR within 10 min hormonal stimulation of AR-positive CAFs (pooled from patients in Table 2). Assembly of this complex is perturbed by addition of 10 nM Rh-2025u or 10 μM enzalutamide (Fig. 6a). As read-out for the complex assembly, we detected a significant release of MMP-2, which was also inhibited by Rh-2025u or enzalutamide (Fig. 6b).

We previously showed that androgen-triggered AR/FlnA/β1 integrin complex controls Tyr 397-FAK phosphorylation, as well as Rac activation, which allow adhesion and motility in fibroblasts^22^. Consistent with our previous findings, the upper section in Fig. 6c shows that 10 min hormone stimulation increases Tyr 397-FAK phosphorylation in lysates from AR-positive CAFs. Such effect is inhibited by Rh-2025u or enzalutamide, while similar amounts of total FAK were detected in the corresponding cell lysates. We then analyzed Rac activation by pull down assay in the same cell lysates (lower section in Fig. 6c). Albeit similar amounts of Rac were loaded, 10 nM R1881 stimulation resulted in a strong increase in Rac-GTP, which was completely blocked by Rh-2025u, as well as enzalutamide. This set of experiments support the conclusion that FAK and Rac activation enables the increase in androgen-induced motility observed in CAFs, as well as the dependence of these processes on AR/FlnA complex assembly.

In conclusion, we posit that the AR/FlnA/β1 integrin-dependent MMP-14 regulation, in combination with increased release of MMP-2, may constitute a signaling circuit relevant to ECM remodeling by androgens in CAFs. On the basis of our previous^21,22^ and present findings, we propose the model depicted in panel d. Androgen stimulation of CAFs rapidly induces the AR/FlnA/β1 integrin complex assembly. This complex recruits and activates FAK and Rac, thereby enabling the basic machinery leading to CAFs migration. The androgen-triggered AR/FlnA/β1 integrin complex also recruits MT-MMP-1, which in turns activates pro-MMP2 and promotes the release of MMP2. Biochemical changes in ECM architecture follow, so that CAFs navigate towards PC cells.

## Discussion

In this paper, we have established a short-term primary culture of CAFs from several PC patients and have shown that AR is expressed at low, but appreciable levels in almost all the CAFs analyzed, even in about 30% of CAFs from PC patients at high (8–10) Gleason’s score. In apparent contrast with these latter findings, a relationship between poor PC outcome and loss of stromal AR has been reported^13,36,58^. This discrepancy might be related to the loss of stromal AR occurring during CAF selection or degradation of the receptor by ubiquitin-proteasome pathways^38^ or epigenetic modifications that destabilize the receptor protein^59^. Noticeably, the number of CAFs from PC patients at intermediate (6–7) Gleason’s score is higher than the one obtained from patients with high (8–10) Gleason’s score (Tables 1), likely because the clinical options in high Gleason’s PC patients are currently controversial^60^.

Since CAFs are unable to secrete prostate specific antigen (PSA), we analyzed the AR transcriptional activity by gene reporter assay and observed a weak AR-mediated gene transcription, regardless of ligand concentration. This latter finding indicates that the absence of reporter response is not caused by a biphasic hormonal effect in our assay. Simultaneously, AR is almost permanently localized in the extra-nuclear compartment of CAFs. Both these properties are likely due to the low amount of receptor protein in CAFs, since scant levels of sex-steroid receptors cannot efficiently undergo dimerization and nuclear translocation (refs. ^20,61^). A transcriptional incompetent AR has been previously detected in CAFs^62^, as well as prostate stromal WPMY-1 cells^63^. Such behavior has been also attributed to the low receptor amounts, which do not yield a sufficient peak numbers in AR-ChIp Seq analysis^64^. By Sanger’s sequencing, no differences were observed between the genomic DNA (gDNA) of the wild type hAR and the receptor expressed in CAFs from patients #9 and #10. Furthermore, direct sequencing of the PCR products showed that AR exon 1 in CAFs contains a normal number of CAG repeats (23;20 repeats, het in patient #9 and 23;27 repeats, het in patient #10). In summary, our biochemical and genomic approaches point each other to the expression of a classic AR in CAFs.

We have then analyzed the rapid, non-genomic receptor functions in CAFs. Ten nanomolar androgens induce within minutes a significant increase in association of AR with FlnA, which correlates with an increase in their extra-nuclear co-localization. The role of FlnA in motility, cytoskeleton remodeling and mechanical-transduction is largely recognized^65^. Relevant to this paper are the studies previously reporting a role for FlnA in androgen/AR signaling. FlnA directly interacts with the hinge-region of AR^66^ and therein refs. A stretch of 334 amino acids of Fln A (Fil_1788−2121_) was firstly identified as interacting with AR and it was also shown that the minimal FlnA domain of interaction lies within a 150-amino acid fragment encompassing repeats 18 and 19 of the filamin IgG-like repeats^67^. AR/FlnA interaction occurs under basal conditions, but is enhanced by the presence of androgens, thereby modulating the transcriptional AR action^45^, the receptor nuclear translocation^67^, as well as the androgen-dependence of LNCaP cells^68^. Our findings subsequently showed that 10 nM androgens significantly enhance extra-nuclear association and co-localization of AR with the full-lenght FlnA in fibroblasts and fibrosarcoma-derived cells. Once assembled, this complex recruits β1 integrin to control activation of Rac and FAK, which coordinately control migration and cell adhesion^22^. Knockdown of FlnA and use of an hAR mutant, unable to interact with FlnA, have supported the idea that AR/FlnA complex acts as a link between androgen signaling, actin cytoskeleton and cell migration in various androgen-responsive cell types^21–24^. The AR/FlnA complex may also affect PC progression and metastasis. Clinical findings have, indeed, correlated the full-length, cytoplasmic FlnA with increased metastatic potential and a hormone-refractory phenotype in PC patients^69^. We here report for the first time that AR/FlnA complex controls the androgen-challenged motility and invasiveness in AR-expressing CAFs. The results here obtained using the stapled peptide, as well as FlnA knockdown corroborate each other in making this complex as a ‘druggable’ target in prostate CAFs.

The role of AR/FlnA complex assembled in CAFs has been analyzed in 3D models of PC. AR-expressing CAFs increase the size of PC organoids and androgens enhance this effect. Perturbing by the peptide the AR/FlnA complex assembled in CAFs results in a reduction of PC organoid size. Findings in PC3 cell organoids strengthen this conclusion, since these cells are AR-negative and unresponsive to androgens. The hypothesis that the stapled peptide acts on PC cell proliferation is excluded by the observation that it does not inhibit the proliferation of serum-treated PC3^21^ or androgen-treated LNCaP cells, even when the cells where cultured in ECM (not shown). Our data rather indicate that the AR/FlnA/β1 integrin complex constitutes a signaling module relevant to ECM remodeling by androgens in CAFs. Perturbation of this complex by the stapled peptide impairs biochemical changes in ECM architecture and migrating ability of CAFs to move towards PC cells. It cannot be excluded, however, that the peptide interferes with other paracrine circuits controlling the stromal-epithelial interactions in PC, thereby affecting the organoid size. In this regard, the NGF/TrkA axis is emerging as a valuable candidate in the reciprocal cross talk between stromal and PC epithelial cells^70^. Preclinical studies, including ours, have highlighted the role of NGF/TrkA signaling in PC progression^24,27,70^. The finding that LNCaP cells release appreciable levels of NGF (Fig. 3S) supports this hypothesis.

Stapled peptides are used to target protein–protein interactions and are designed to stabilize and constrain an α-helical structure^71,72^. These compounds exhibit drug-like properties (i.e., enhanced cell permeability, resistance to proteolytic degradation)^72^ and most of them have entered clinical trials^72,73^. The stapled peptide here used derives from the AR 622–670 amino acid sequence responsible for interaction of the receptor with FlnA^22,45^ and exhibits some properties that should make the compound very specific and safe ‘in vivo*’*. Its use in prostate CAFs would improve the therapeutic options in PC.

CAFs are more genetically stable and less likely to acquire therapy resistance than tumor cells^4^. They have emerged in recent years as a promising diagnostic and therapeutic target in different cancer models. Clinical trials have recently assessed the therapeutic value of human CD8+ T cells engineered to target FAP-expressing cells (such as CAFs), as well as two FAP-antibody/cytokine fusion proteins, RO6874281 and RO6874813^4^ and therein refs. Further, the diagnostic and therapeutic value of radio-immunoconjugates and radio-tracers that bind to FAP with high affinity and are rapidly internalized in cells expressing high levels of FAP, such as CAFs, has been validated in vitro and in vivo^74^. Precision strategies have been developed to target CAFs, using FAP-ligands coupled to cytotoxic drugs^75^ and imaging of tumor stroma based on FAP-targeted radiotracers is emerging as a powerful tool in diagnosis and follow-up of human cancer^76^. Similar approaches might be exploited to deliver the stapled peptide in prostate CAFs. Noticeably, most of the clinical trials so far used to trigger stromal components improve, or even allow drug delivery to cancer cells, and most of the drugs commonly used in cancer therapy simultaneously act on both cancer cells and CAFs. Approaches that exclusively target cancer stroma are still lacking^4^.

In conclusion, this study identifies the relevance of AR/FlnA complex as a new biomarker in prostate CAFs. Its identification in CAFs would represent a valuable approach to characterize the molecular signature of PC microenvironment and evaluate the benefit of new drugs (peptides/stapled peptides) suitable for diagnostic and therapeutic tools in PC.

## Supplementary information

Supplemental Data

Table I S

Table II S

Table III S

Figure 1S

Figure 2S

Figure 3S

Figure 4S

## References

[1] Pignot G (2018). Systemic treatments for high-risk localized prostate cancer. Nat. Rev. Urol..

[2] Evans AJ (2018). Treatment effects in prostate cancer. Mod. Pathol..

[3] Attard G, Antonarakis ES (2016). Prostate cancer: AR aberrations and resistance to abiraterone or enzalutamide. Nat. Rev. Urol..

[4] Sahai E (2020). A framework for advancing our understanding of cancer-associated fibroblasts. Nat. Rev. Cancer.

[5] Olumi AF (1999). Carcinoma-associated fibroblasts direct tumor progression of initiated human prostatic epithelium. Cancer Res..

[6] Hayward SW (2001). Malignant transformation in a nontumorigenic human prostatic epithelial cell line. Cancer Res..

[7] Taylor RA, Risbridger GP (2008). Prostatic tumor stroma: a key player in cancer progression. Curr. Cancer Drug Targets.

[8] Giannoni E, Bianchini F, Calorini L, Chiarugi P (2011). Cancer associated fibroblasts exploit reactive oxygen species through a proinflammatory signature leading to epithelial mesenchymal transition and stemness. Antioxid. Redox Signal..

[9] Fiaschi T (2012). Reciprocal metabolic reprogramming through lactate shuttle coordinately influences tumor-stroma interplay. Cancer Res..

[10] Cabarcas SM, Mathews LA, Farrar WL (2011). The cancer stem cell niche–there goes the neighborhood?. Int. J. Cancer.

[11] Henshall SM (2001). Altered expression of androgen receptor in the malignant epithelium and adjacent stroma is associated with early relapse in prostate cancer. Cancer Res..

[12] Ricciardelli C (2005). Androgen receptor levels in prostate cancer epithelial and peritumoral stromal cells identify non-organ confined disease. Prostate.

[13] Wikström P, Marusic J, Stattin P, Bergh A (2009). Low stroma androgen receptor level in normal and tumor prostate tissue is related to poor outcome in prostate cancer patients. Prostate.

[14] Yu S (2013). Androgen receptor in human prostate cancer-associated fibroblasts promotes prostate cancer epithelial cell growth and invasion. Med. Oncol..

[15] Castoria G, Auricchio F, Migliaccio A (2017). Extranuclear partners of androgen receptor: at the crossroads of proliferation, migration, and neuritogenesis. FASEB J..

[16] Lai K-P, Yamashita S, Huang C-K, Yeh S, Chang C (2012). Loss of stromal androgen receptor leads to suppressed prostate tumourigenesis via modulation of pro-inflammatory cytokines/chemokines. EMBO Mol. Med..

[17] Ricke EA (2012). Androgen hormone action in prostatic carcinogenesis: stromal androgen receptors mediate prostate cancer progression, malignant transformation and metastasis. Carcinogenesis.

[18] Montgomery RB (2008). Maintenance of intratumoral androgens in metastatic prostate cancer: a mechanism for castration-resistant tumor growth. Cancer Res..

[19] Leach DA (2015). Stromal androgen receptor regulates the composition of the microenvironment to influence prostate cancer outcome. Oncotarget.

[20] Castoria G (2003). Androgen-stimulated DNA synthesis and cytoskeletal changes in fibroblasts by a nontranscriptional receptor action. J. Cell Biol..

[21] Castoria G (2014). Role of non-genomic androgen signalling in suppressing proliferation of fibroblasts and fibrosarcoma cells. Cell Death Dis..

[22] Castoria G (2011). Androgen-induced cell migration: role of androgen receptor/filamin A association. PLoS ONE.

[23] Di Donato M (2015). Cross-talk between androgen receptor/filamin A and TrkA regulates neurite outgrowth in PC12 cells. Mol. Biol. Cell.

[24] Di Donato M, Cernera G, Auricchio F, Migliaccio A, Castoria G (2018). Cross-talk between androgen receptor and nerve growth factor receptor in prostate cancer cells: implications for a new therapeutic approach. Cell Death Discov..

[25] Chang CS, Kokontis J, Liao ST (1988). Molecular cloning of human and rat complementary DNA encoding androgen receptors. Science.

[26] Verrijdt G (2000). Change of specificity mutations in androgen-selective enhancers. Evidence for a role of differential DNA binding by the androgen receptor. J. Biol. Chem..

[27] Di Donato M, Cernera G, Migliaccio A, Castoria G (2019). Nerve growth factor induces proliferation and aggressiveness in prostate cancer cells. Cancers.

[28] Pagano M (2004). Differentiation of H9c2 cardiomyoblasts: the role of adenylate cyclase system. J. Cell. Physiol..

[29] Giovannelli P, Di Donato M, Auricchio F, Castoria G (2014). Analysis of the androgen receptor/filamin a complex in stromal cells. Methods Mol. Biol..

[30] Giovannelli P, Di Donato M, Auricchio F, Castoria G, Migliaccio A (2019). Androgens induce invasiveness of triple negative breast cancer cells through AR/Src/PI3-K complex assembly. Sci. Rep..

[31] Chua CW (2014). Single luminal epithelial progenitors can generate prostate organoids in culture. Nat. Cell Biol..

[32] Beshiri ML (2018). A PDX/organoid biobank of advanced prostate cancers captures genomic and phenotypic heterogeneity for disease modeling and therapeutic screening. Clin. Cancer Res..

[33] Drost J (2016). Organoid culture systems for prostate epithelial and cancer tissue. Nat. Protoc..

[34] Rossi V (2019). Estrogens modulate somatostatin receptors expression and synergize with the somatostatin analog pasireotide in prostate cells. Front Pharm..

[35] Kalluri R, Zeisberg M (2006). Fibroblasts in cancer. Nat. Rev. Cancer.

[36] Leach, D. A. & Buchanan, G. Stromal androgen receptor in prostate cancer development and progression. *Cancers* **9**, 10 (2017). 10.3390/cancers9010010 PMC5295781 28117763

[37] Wilson CM, McPhaul MJ (1994). A and B forms of the androgen receptor are present in human genital skin fibroblasts. Proc. Natl Acad. Sci. USA.

[38] Lin H-K (2002). Phosphorylation-dependent ubiquitylation and degradation of androgen receptor by Akt require Mdm2 E3 ligase. EMBO J..

[39] Cardozo CP (2003). C-terminal Hsp-interacting protein slows androgen receptor synthesis and reduces its rate of degradation. Arch. Biochem. Biophys..

[40] Burska UL (2013). Deubiquitinating enzyme Usp12 is a novel co-activator of the androgen receptor. J. Biol. Chem..

[41] Slavin S (2014). Estrogen receptor α in cancer-associated fibroblasts suppresses prostate cancer invasion via modulation of thrombospondin 2 and matrix metalloproteinase 3. Carcinogenesis.

[42] Di Zazzo E, Galasso G, Giovannelli P, Di Donato M, Castoria G (2018). Estrogens and their receptors in prostate cancer: therapeutic implications. Front. Oncol..

[43] Brinkmann AO (1986). Characterization of androgen receptors after photoaffinity labelling with [3H]methyltrienolone (R1881). J. Steroid Biochem..

[44] Tran C (2009). Development of a second-generation antiandrogen for treatment of advanced prostate cancer. Science.

[45] Loy CJ, Sim KS, Yong EL (2003). Filamin-A fragment localizes to the nucleus to regulate androgen receptor and coactivator functions. PNAS.

[46] Verdine GL, Hilinski GJ (2012). Stapled peptides for intracellular drug targets. Meth. Enzymol..

[47] Chatterjee P (2019). Supraphysiological androgens suppress prostate cancer growth through androgen receptor–mediated DNA damage. J. Clin. Invest..

[48] Green SM, Mostaghel EA, Nelson PS (2012). Androgen action and metabolism in prostate cancer. Mol. Cell. Endocrinol..

[49] Cioni B (2018). Loss of androgen receptor signaling in prostate cancer-associated fibroblasts (CAFs) promotes CCL2- and CXCL8-mediated cancer cell migration. Mol. Oncol..

[50] Geldof AA, Van Haarst EP, Newling DWW (1998). Neurotrophic factors in prostate and prostatic cancer. Prostate Cancer Prostatic Dis..

[51] Delsite R, Djakiew D (1999). Characterization of nerve growth factor precursor protein expression by human prostate stromal cells: a role in selective neurotrophin stimulation of prostate epithelial cell growth. Prostate.

[52] Silver RI (1994). Cell type specific expression of steroid 5α-reductase 2. J. Urol..

[53] Moore CB, Guthrie EH, Huang MT-H, Taxman DJ (2010). Short hairpin RNA (shRNA): design, delivery, and assessment of gene knockdown. Methods Mol. Biol..

[54] Ridley AJ (2003). Cell migration: integrating signals from front to back. Science.

[55] Gong Y, Chippada-Venkata UD, Oh WK (2014). Roles of matrix metalloproteinases and their natural inhibitors in prostate cancer progression. Cancers.

[56] Nguyen H-L (2011). Oxidative stress and prostate cancer progression are elicited by membrane-type 1 matrix metalloproteinase. Mol. Cancer Res..

[57] Mori H (2013). Transmembrane/cytoplasmic, rather than catalytic, domains of Mmp14 signal to MAPK activation and mammary branching morphogenesis via binding to integrin β1. Development.

[58] Olapade-Olaopa EO (1999). Malignant transformation of human prostatic epithelium is associated with the loss of androgen receptor immunoreactivity in the surrounding stroma. Clin. Cancer Res..

[59] Keil KP (2014). Androgen receptor DNA methylation regulates the timing and androgen sensitivity of mouse prostate ductal development. Dev. Biol..

[60] Saito, S., Ito, K., Yorozu, A. & J-POPS Study Group Steering Committee. Weighing up prostate cancer treatment outcomes. *Nature* **574**, S84 (2019).

[61] Vallejo G, Ballaré C, Barañao JL, Beato M, Saragüeta P (2005). Progestin activation of nongenomic pathways via cross talk of progesterone receptor with estrogen receptor beta induces proliferation of endometrial stromal cells. Mol. Endocrinol..

[62] Ellem SJ (2014). A pro-tumourigenic loop at the human prostate tumour interface orchestrated by oestrogen, CXCL12 and mast cell recruitment. J. Pathol..

[63] Singh M (2014). Stromal androgen receptor in prostate development and cancer. Am. J. Pathol..

[64] Nash, C. et al. Genome-wide analysis of androgen receptor binding and transcriptomic analysis in mesenchymal subsets during prostate development. *Dis. Models Mechan.* **12**, dmm039297 (2019). 10.1242/dmm.039297 PMC6679388 31350272

[65] Zhou A-X, Hartwig JH, Akyürek LM (2010). Filamins in cell signaling, transcription and organ development. Trends Cell Biol..

[66] Savoy RM, Ghosh PM (2013). The dual role of filamin A in cancer: can’t live with (too much of) it, can’t live without it. Endocr. Relat. Cancer.

[67] Ozanne DM (2000). Androgen receptor nuclear translocation is facilitated by the f-actin cross-linking protein filamin. Mol. Endocrinol..

[68] Wang Y (2007). A 90 kDa fragment of filamin A promotes Casodex-induced growth inhibition in Casodex-resistant androgen receptor positive C4-2 prostate cancer cells. Oncogene.

[69] Bedolla RG (2009). Nuclear versus cytoplasmic localization of filamin A in prostate cancer: immunohistochemical correlation with metastases. Clin. Cancer Res..

[70] Singh R (2019). TRAF4-mediated ubiquitination of NGF receptor TrkA regulates prostate cancer metastasis. J. Clin. Invest..

[71] Higueruelo AP, Jubb H, Blundell TL (2013). Protein-protein interactions as druggable targets: recent technological advances. Curr. Opin. Pharm..

[72] Wittrup KD, Verdine GL (2012). Protein engineering for therapeutics, Part A. Preface. Methods Enzymol..

[73] Sorolla A (2020). Precision medicine by designer interference peptides: applications in oncology and molecular therapeutics. Oncogene.

[74] Fischer E (2012). Radioimmunotherapy of fibroblast activation protein positive tumors by rapidly internalizing antibodies. Clin. Cancer Res..

[75] Kim, M.-G., Shon, Y., Kim, J. & Oh, Y.-K. Selective activation of anticancer chemotherapy by cancer-associated fibroblasts in the tumor microenvironment. *J. Natl Cancer Inst.* **109**, djw186 (2016). PMID: 27615014; PMCID: PMC6284259. 10.1093/jnci/djw186 PMC6284259 27615014

[76] Loktev A (2018). A tumor-imaging method targeting cancer-associated fibroblasts. J. Nucl. Med..

