# Correction to: The androgen receptor/filamin A complex as a target in prostate cancer microenvironment

**DOI:** 10.1038/s41419-021-03483-4

**Published:** 2021-03-15

**Authors:** Marzia Di Donato, Alice Zamagni, Giovanni Galasso, Erika Di Zazzo, Pia Giovannelli, Maria Vittoria Barone, Michele Zanoni, Roberta Gunelli, Matteo Costantini, Ferdinando Auricchio, Antimo Migliaccio, Anna Tesei, Gabriella Castoria

**Affiliations:** 1 Department of Precision Medicine, University of Campania ‘L. Vanvitelli’- Via L. De Crecchio, 7- 80138 Naples, Italy; 2 Biosciences Laboratory, Istituto Scientifico Romagnolo per lo Studio e la Cura dei Tumori (IRCCS, Via P. Maroncelli, 40-47014 Meldola, Forlì Italy; 3 Department of Translational Medical Science, University of Naples ‘Federico II’- Via S. Pansini, 5- 80131 Naples, Italy; 4 Department of Urology, Morgagni Pierantoni Hospital, Forli, Italy; 5 Pathology Unit, Morgagni Pierantoni Hospital, Forlì, Italy

**Keywords:** Prostate cancer, Cancer microenvironment

Correction to: *Cell Death & Disease*

10.1038/s41419-021-03402-7 published online 26 January 2021

The original version of this article unfortunately contained a mistake. Roberta Gunelli is affiliated with the Department of Urology, Morgagni Pierantoni Hospital, Forli, Italy and Matteo Costantini is affiliated with Pathology Unit, Morgagni Pierantoni Hospital, Forlì, Italy. The authors apologize for the mistake. The original article has been corrected.

