# Supplementary material for: The androgen receptor/filamin A complex as a target in prostate cancer microenvironment
Source: Cell Death Dis. 2021 Jan 26;12(1):127. doi: 10.1038/s41419-021-03402-7 (PMC7838283; doi:10.1038/s41419-021-03402-7)
Supplement: Supplementary file 1 — Supplemental Data [file 41419_2021_3402_MOESM1_ESM.docx]

**SUPPLEMENTAL DATA**

**SUPPLEMENTAL FIGURES.**

**Fig. 1S. Prostate CAFs express AR.**

CAF lysate proteins from patients # 1-17 (Table II in the main text) were prepared. Panels **a**, **b** and **c** show the WB of lysate proteins using antibodies against the indicated proteins. Lysate proteins from LNCaP or MCF-7 or T47D cells were analyzed in parallel. Molecular weights are indicated.

**Fig. 2S. The stapled peptide, Rh2025u specifically affects the androgen-triggered responses in AR-expressing PC-derived cells.**

A schematic representation of the modified, stapled Rh2025u peptide is shown in panel **a.**

**The peptide does not affect migration and invasiveness induced by serum in AR-positive CAFs from different patients (b).** Quiescent AR-positive CAFs from the indicated patients (Table II in the main text) were challenged with 20% serum, in the absence or presence of 10 nM Rh2025u (Rh). CAFs were allowed to migrate or invade for 7h or 24h, respectively and counted as described in Methods. Data are expressed as fold increase. Means are shown.

**The peptide does not affect migration induced by serum in PC-derived cells (c).** Quiescent LNCaP, DU-145 or PC3 cells were challenged with 20% serum, in the absence or presence of 10 nM Rh2025u (Rh), then allowed to migrate for 7h and counted as described in Methods. Data are expressed as fold increase. Means and SEMs are shown.

**The peptide does not affect migration and invasiveness induced by serum in AR-negative CAFs from different patients (d).** Quiescent AR-negative CAFs from the indicated patients (Table II in the main text) were challenged with 20% serum, in the absence or presence of 10 nM Rh2025u (Rh), then allowed to migrate or invade for 7h or 24h, respectively and counted as described in Methods. Data are expressed as fold increase. Means are shown.

**The peptide does not affect the androgen-triggered gene transcription in LNCaP cells (e).** LNCaP cells were transfected with 3416-luc construct, as described in the main text (Methods section). Transfected cells were made quiescent and then left unstimulated or stimulated for 24h with 10 nM R1881, in the absence or presence of 10 nM Rh2025u. Luciferase activity was assayed, normalized using β-gal as an internal control and expressed as fold induction. Means are shown. *n*, represents the number of experiments.

**Fig. 3 S. AR-positive CAFs from PC patients increase the size of LNCaP organoids: effect of DHT and low R1881 concentration (a** and **b).** AR-positive CAFs from patients in Table II (main text) were pooled together, then co-cultured with LNCaP cells stably expressing GFP (GFP-LNCaP cells) in ECM. In **a**, a 3D structure was observed in GFP-LNCaP cells after 3 days of co-culture with CAFs. At that time, the organoids were left untreated or treated with 2 nM R1881 or 10 nM DHT. Changes in dimension and structure of organoids were monitored for additional 15 days. Phase-contrast microscopy (Fig. 3S **a**), IF images (Fig. 3S **a**) and quantification of data (Fig. 3S **b**) are shown. The results support the conclusion that stimulation with 10 nM DHT significantly increased the LNCaP organoid size. A slight, but significant stimulation was observed by using 2 nM R1881.

**Experiments in 2D co-culture using AR-negative CAFs (c).** AR-negative CAFs from patients in Table II (main text) were pooled and then co-cultured in 2D with LNCaP cells, as reported in Methods. AR-negative CAFs were co-cultured with LNCaP cells or unconditioned medium (ctrl medium), using a Trans-well system. Cells were left untreated or treated with 10 nM R1881. CAFs were allowed to invade for 16 h, stained and counted as described in Methods. Data are expressed as fold increase in number of invading CAFs. ***n*** represents the number of experiments. Means and SEM are shown. Data in Fig. 3S **c** show that co-culture with LNCaP cells slightly increased (from 1 to 1,35) the number of invading AR-negative CAFs, as compared to the control, unconditioned medium. Expectedly, AR-negative CAFs were insensitive to R1881 (Fig. 3S **c**).

**ELISA assays for androgens, EGF and NGF released by PC-derived cells (d).**

LNCaP, DU-145 and PC3 cells were made quiescent. Conditioned media were collected after 48h and analysed by ELISA kits, as reported in Methods. Panels in **d** show that albeit at different extent, conditioned media from LNCaP, PC3 and DU-145 cells contain androgens or EGF or NGF. Specifically, LNCaP cells release 0,05 nM NGF, which is enough to induce almost the 50% of the NGF-receptor activation ([Maliartchouk](https://www.ncbi.nlm.nih.gov/pubmed/?term=Maliartchouk%20S%5BAuthor%5D&cauthor=true&cauthor_uid=9236214) &  [Saragovi](https://www.ncbi.nlm.nih.gov/pubmed/?term=Saragovi%20HU%5BAuthor%5D&cauthor=true&cauthor_uid=9236214), J. Neurosci. 1997, 17, 6031).^,^LNCaP cells also release appreciable testosterone levels, which might be coverted into DHT by stromal 5-alpha reductase enzymes. These findings might account for the AR-positive CAFs recruitment by LNCaP cells under basal conditions (see also Fig. 3 in the main text). PC3 cells prevalently release the androgens, testosterone and DHT, together with a very small amount of EGF. Noticeably, the amount of secreted EGF is too low to exert biological effects. EGF, indeed, stimulates the optimal mitogenesis at levels ranging from 0.5 to 2 ng/ml (Wennstrom & Downward, Mol Cell Biol. 1999, 19, 4279) and cell motility at levels ranging from 100 to 150 ng/ml (Chen et al., J Cell Biol. 1994, 124, 547). The release of androgens by PC3 cells might account for the basal recruitment of AR-positive CAFs by these cells. At last, DU-145 cells release traceable amounts of NGF and testosterone, which can weakly mediate the recruitment of AR-positive CAFs.

**Fig. 4S. FlnA expression, AR/FlnA co-localization, migration and invasion in CAFs.**

**Prostate CAFs express FlnA (a).** Upper section shows the expression of FlnA revealed by WB of CAF lysate proteins derived from the indicated patients (Table II in the main text). Lower section shows the WB of corresponding lysate proteins using the anti-tubulin antibody, as loading control.

**R1881 increases the co-localization ratio between AR and FlnA in CAFs (b).** Quiescent CAFs obtained from the indicated patients (Table II in the main text) were plated on coverslips and made quiescent. Cells were left unchallenged or challenged for 10 min with 10 nM R1881. AR and FlnA were stained as described in Methods and AR/FlnA co-localization was analyzed by confocal microscopy. Quantitative analysis in CAFs from the indicated patients shows that androgen increases AR/FlnA co-localization ratio by almost 2-fold. Data derive from 3 different experiments. Means and SEM are shown.

**FlnA staining is specific (c).** CAFs from patient # 4 were plated on coverslips and then made quiescent. Cells were then challenged for 10 min with 10 nM R1881 and stained with the secondary antibody in combination with the FITC-conjugated anti AR antibody. CAFs on coverslips were analyzed by confocal microscopy and representative images were captured and shown as control.

**Migration (d) and invasiveness (e) of CAFs: dependence on androgen concentration.** Quiescent CAFs from patient #17 (Table II in the main text) were challenged with the indicated concentrations of R1881 and DHT**.** Cells were allowed to migrate (**d**) or invade (**e**) for 7h or 24h, respectively and then counted as described in Methods. Data are expressed as fold increase. Means and SEMs are shown. ***n*** represents the number of experiments. *****p< 0,01, for the indicated experimental points *versus* the corresponding untreated cells.

**SUPPLEMENTAL TABLES**

**Table 1S. The Rh-2025u peptide reverses migration and invasiveness induced by R1881 in CAFs.**

Quiescent CAFs from the indicated patients were left unstimulated or stimulated with 10 nM R1881, in the absence or presence of 10 nM Rh-2025u peptide (Rh). Cells were allowed to migrate or invade for 7 or 24 h, respectively. Migrating or invading cells were counted, as described in Methods. Data are expressed as fold increase in number of migrated or invaded cells.

**Table 2S. AR is prevalently localized in cytoplasm of CAFs.**

CAFs from the indicated patients were plated on coverslips and made quiescent. Cells were left untreated or treated for 30 min with 10 nM R1881. AR and nuclei were stained, as described in the main text. The intracellular receptor localization was analyzed by IF. Cells predominantly exhibiting cytoplasmic or nuclear-cytoplasmic AR staining were scored and expressed as a percentage of total cells. Data were averaged from 3 independent experiments. Means and SEM are shown.

**Table 3S. AR weakly mediates the androgen-induced gene transcription in CAFs.**

CAFs from the indicated patients were transfected with 3416-luc (ARE-luc) encoding plasmid, as described in the main text. Quiescent cells were then left unstimulated or stimulated for 18 h with 10 nM R1881. When indicated, CAFs were challenged with 1 nM or 10 nM R1881. Luciferase activity was assayed, normalized using β-gal as an internal control, and expressed as fold induction. Data were averaged from two independent experiments. Means are shown.
