# Supplementary material for: The androgen receptor/filamin A complex as a target in prostate cancer microenvironment
Source: Cell Death Dis. 2021 Jan 26;12(1):127. doi: 10.1038/s41419-021-03402-7 (PMC7838283; doi:10.1038/s41419-021-03402-7)
Supplement: Supplementary file 2 — Table I S [file 41419_2021_3402_MOESM2_ESM.doc]

|  | **Migration**  **(fold Increase)** | **Migration**  **(fold Increase)** | **Invasion**  **(fold increase)** | **Invasion**  **(fold increase)** |
| --- | --- | --- | --- | --- |
| **Patient** | **R1881** | **R1881 + Rh** | **R1881** | **R1881 + Rh** |
| **#1** | 2,26 | 0,9 | 2,5 | 1 |
| **#2** | 1,7 | 0,93 | 1,8 | 0,77 |
| **#3** | 2,1 | 0,8 | 2,27 | 0,88 |
| **#5** | 1,88 | 0,76 | 1,9 | 1 |
| **#6** | 2,1 | 0,8 | 2 | 0,77 |
| **#7** | 1,6 | 0,46 | 1,78 | 0,99 |
| **#9** | 2,1 | 1 | 1,99 | 0,9 |
| **#10** | 2,05 | 0,88 | 2,13 | 1 |
| **#15** | 1,4 | 0,99 | 1,9 | 0,82 |
| **#16** | 1,8 | 0,8 | 1,7 | 0,8 |
| **#17** | 2,5 | 1,1 | 2,4 | 0,8 |

**Table I S. Androgen-triggered migration and invasion in CAFs from PC patient: the inhibitory effect of Rh 2025u peptide**
