# Supplementary material for: The androgen receptor/filamin A complex as a target in prostate cancer microenvironment
Source: Cell Death Dis. 2021 Jan 26;12(1):127. doi: 10.1038/s41419-021-03402-7 (PMC7838283; doi:10.1038/s41419-021-03402-7)
Supplement: Supplementary file 3 — Table II S [file 41419_2021_3402_MOESM3_ESM.doc]

**Table II S. AR localization in CAFs from PC patients**

| **cytoplasmic AR**  **(% of cells)** | | | **nucleo/cytoplasmic AR**  **(% of cells)** | |
| --- | --- | --- | --- | --- |
| **Patient** | **- R1881** | **+ R1881** | **- R1881** | **+ R1881** |
| #1 | 83±2 | 79±2 | 17±2 | 21±2 |
| #2 | 87±3 | 80±2 | 13±3 | 20±2 |
| #3 | 88±1 | 83±2 | 12±1 | 17±2 |
| #4 | 80±4 | 76±3 | 20±4 | 24±3 |
| #5 | 84±3 | 81±3 | 16±3 | 19±3 |
| #6 | 88±1 | 84±2 | 12±1 | 16±2 |
| #7 | 82±3 | 78±2 | 18±3 | 22±2 |
