# Supplementary material for: The androgen receptor/filamin A complex as a target in prostate cancer microenvironment
Source: Cell Death Dis. 2021 Jan 26;12(1):127. doi: 10.1038/s41419-021-03402-7 (PMC7838283; doi:10.1038/s41419-021-03402-7)
Supplement: Supplementary file 4 — Table III S [file 41419_2021_3402_MOESM4_ESM.doc]

**Table III S. The androgen-induced AR transactivation in CAFs from PC patients.**

| **Patient** | **Androgen-stimulated ARE-luc**  **(fold induction)** |
| --- | --- |
| 1 | 1,3 |
| 2 | 1,5 |
| 3 | 1,6 |
| 4 | 1,2 |
| 5 | 1,3 |
| 6 | 1,7 |
| 7 | 1,01 |
| 15 | 1,4 |
| 17 | 1,5 |

| **Patient** | **Androgen-stimulated ARE-luc**  **(fold induction)** |
| --- | --- |
|  | 1 nM R1881 10 nM R1881 |
| 16 | 1,64 1,92 |
