# Supplementary figures and images for: The androgen receptor/filamin A complex as a target in prostate cancer microenvironment
Source: Cell Death Dis. 2021 Jan 26;12(1):127. doi: 10.1038/s41419-021-03402-7 (PMC7838283; doi:10.1038/s41419-021-03402-7)

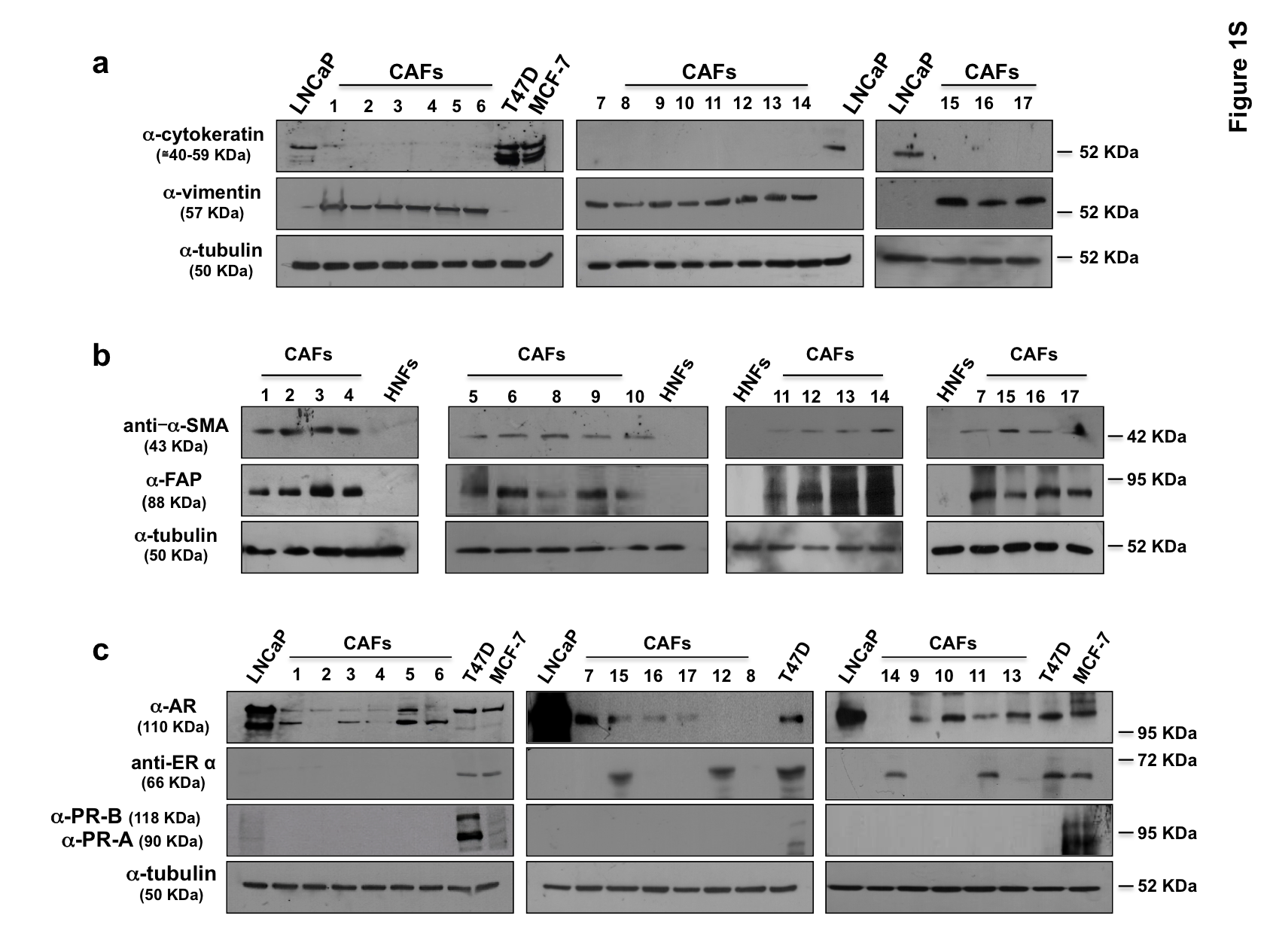

Supplement: Supplementary file 5 — Figure 1S [file 41419_2021_3402_MOESM5_ESM.tif]

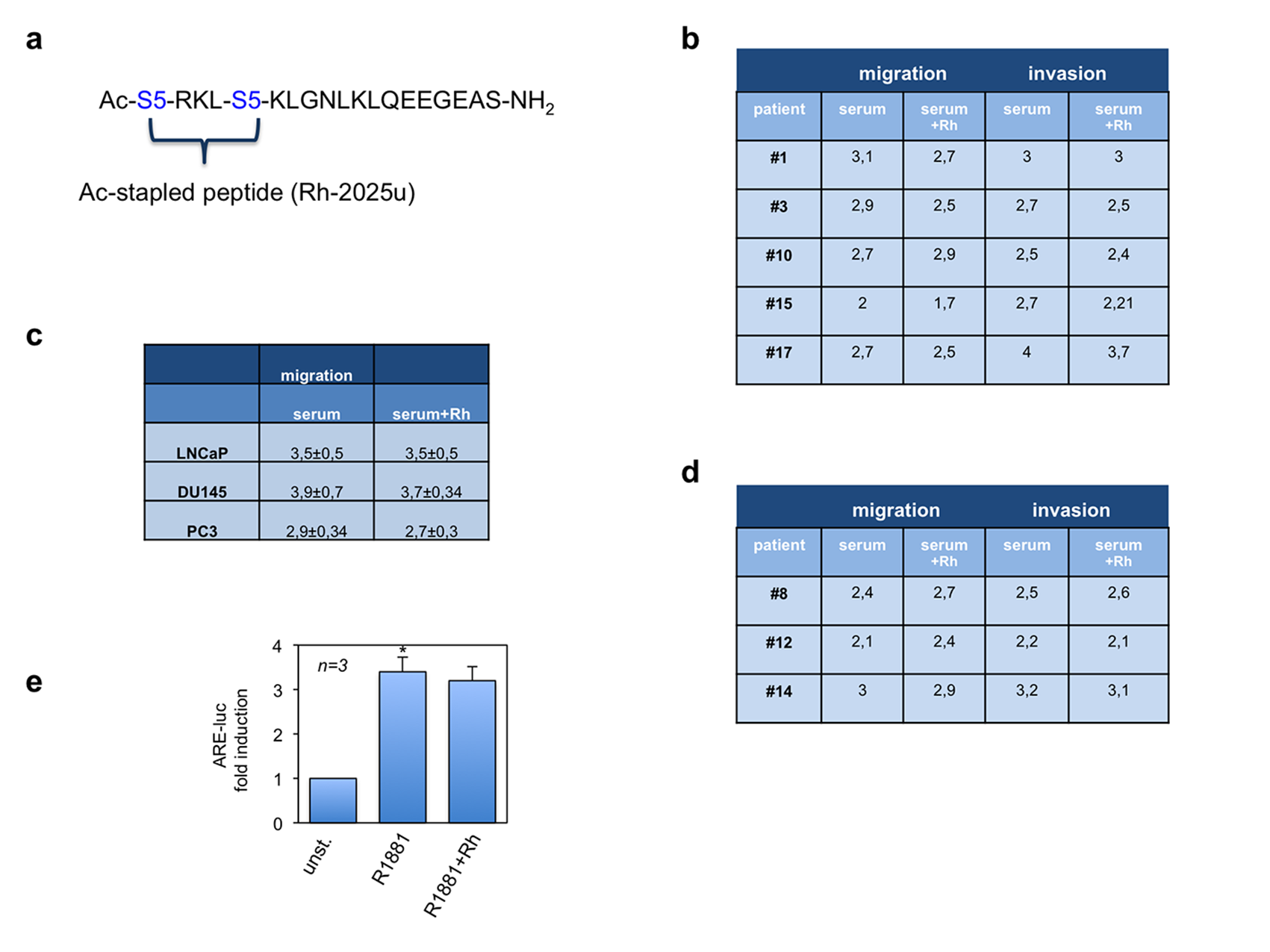

Supplement: Supplementary file 6 — Figure 2S [file 41419_2021_3402_MOESM6_ESM.tif]

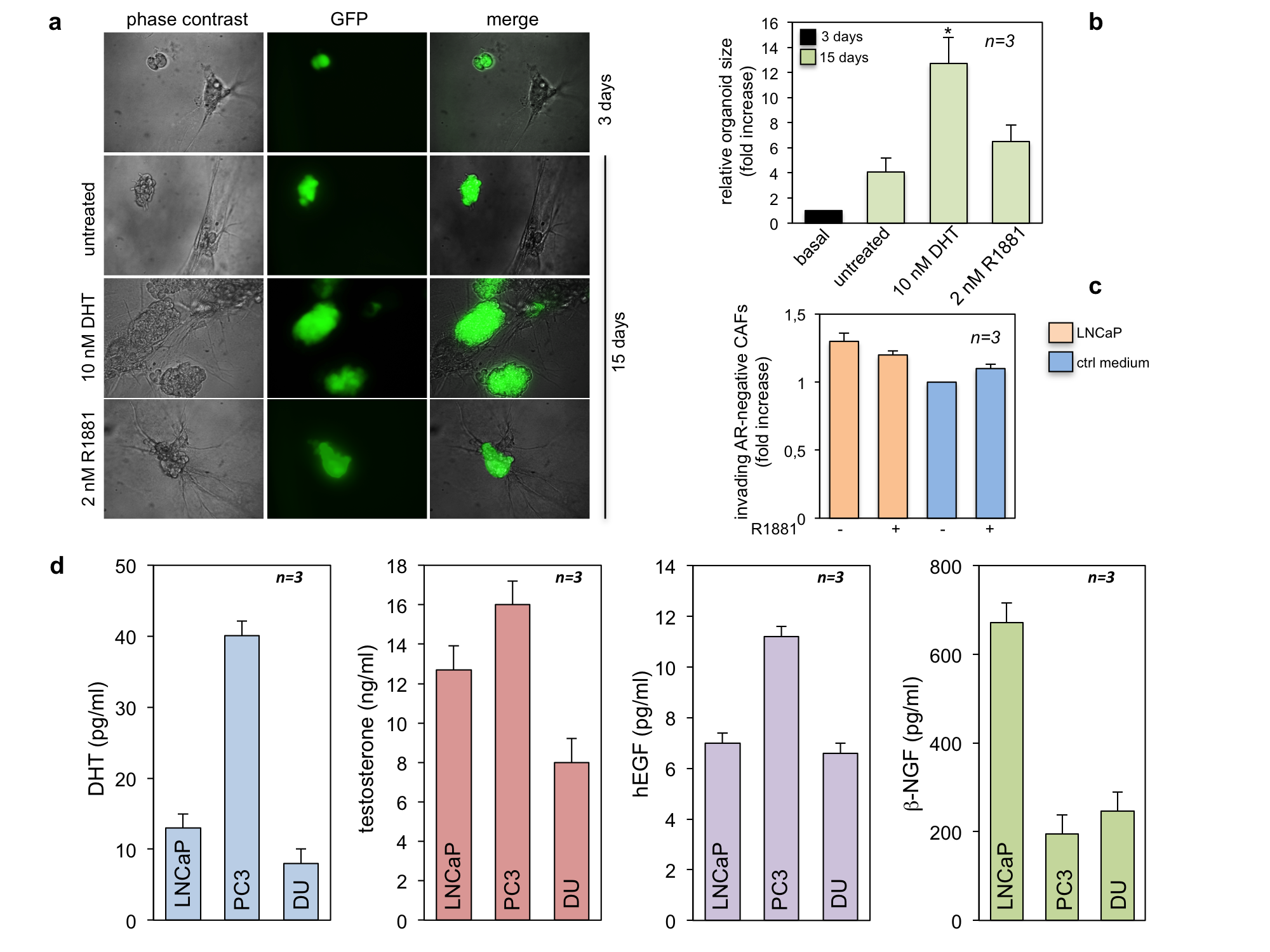

Supplement: Supplementary file 7 — Figure 3S [file 41419_2021_3402_MOESM7_ESM.tif]

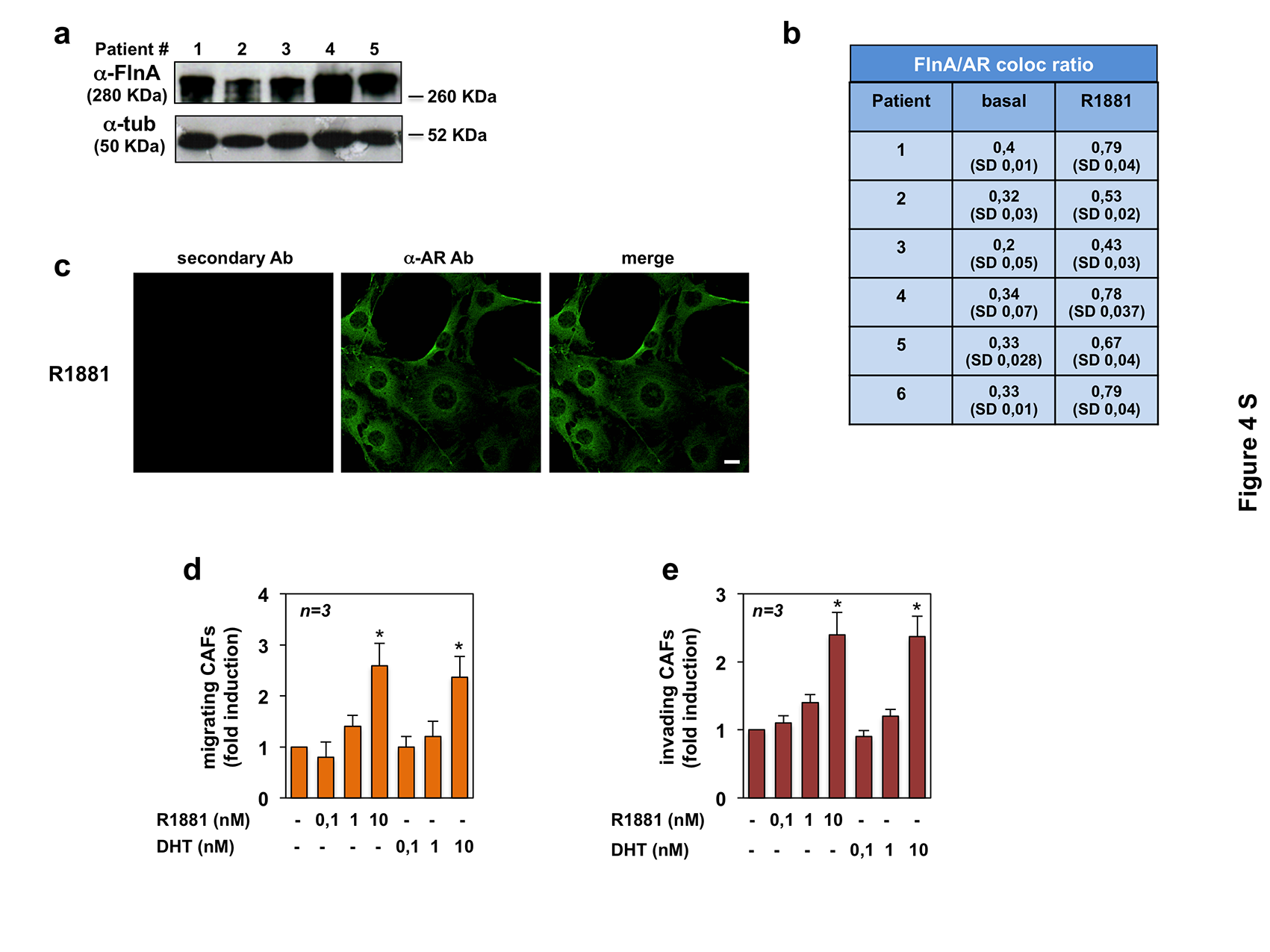

Supplement: Supplementary file 8 — Figure 4S [file 41419_2021_3402_MOESM8_ESM.tif]
